# Human-muscle-inspired single fibre actuator with reversible percolation

**DOI:** 10.1038/s41565-022-01220-2

**Published:** 2022-10-27

**Authors:** In Ho Kim, Subi Choi, Jieun Lee, Jiyoung Jung, Jinwook Yeo, Jun Tae Kim, Seunghwa Ryu, Suk-kyun Ahn, Jiheong Kang, Philippe Poulin, Sang Ouk Kim

**Affiliations:** 1 Department of Materials Science and Engineering, Korea Advanced Institute of Science and Technology (KAIST), Daejeon, Republic of Korea; 2 National Creative Research Initiative Center for Multi-dimensional Directed Nanoscale Assembly, Korea Advanced Institute of Science and Technology (KAIST), Daejeon, Republic of Korea; 3 Department of Polymer Science and Engineering, Pusan National University, Busan, Republic of Korea; 4 Department of Mechanical Engineering, Korea Advanced Institute of Science and Technology (KAIST), Daejeon, Republic of Korea; 5 Université de Bordeaux, CNRS, Centre de Recherche Paul Pascal, Pessac, France; 6 Materials Creation, Seoul, Republic of Korea

**Keywords:** Actuators, Mechanical and structural properties and devices, Molecular self-assembly

## Abstract

Artificial muscles are indispensable components for next-generation robotics capable of mimicking sophisticated movements of living systems. However, an optimal combination of actuation parameters, including strain, stress, energy density and high mechanical strength, is required for their practical applications. Here we report mammalian-skeletal-muscle-inspired single fibres and bundles with large and strong contractive actuation. The use of exfoliated graphene fillers within a uniaxial liquid crystalline matrix enables photothermal actuation with large work capacity and rapid response. Moreover, the reversible percolation of graphene fillers induced by the thermodynamic conformational transition of mesoscale structures can be in situ monitored by electrical switching. Such a dynamic percolation behaviour effectively strengthens the mechanical properties of the actuator fibres, particularly in the contracted actuation state, enabling mammalian-muscle-like reliable reversible actuation. Taking advantage of a mechanically compliant fibre structure, smart actuators are readily integrated into strong bundles as well as high-power soft robotics with light-driven remote control.

## Abstract (web summary)

The reversible percolation of a graphene filler network in a liquid crystalline elastomer matrix enables the realization of mammalian-muscle-like actuation.

## Main

Artificial muscles undergo repeated cycles of geometric or dimensional modulation on external stimuli, holding great potential in next-generation soft robotics^1,2^, smart clothing^3^, bioinspired systems^4,5^, implantable medical reinforcements^6^ and so on. Among several different actuating characteristics for artificial muscles^7–10^, stimuli-responsive polymer-based structures^11–18^ having light weight, high elasticity and manageable processability are expected to address the intrinsic limitations of traditional hydraulic and pneumatic actuators. Unfortunately, these polymeric materials with large macroscopic shape change commonly suffer from the inherent vulnerable mechanical properties. In this regard, composite structures incorporating rigid fillers into tailored polymer matrices are attracting research attention as a route to achieve high mechanical properties, concurrent large actuation deformation and other functionalities^11–13,19–21^. The principal research direction for such composite-type actuators has focused on the optimal material choice and post-synthetic macroscopic structural engineering, such as helical twisting or coiling, aiming at the maximal amplification of actuation performances thus far. Nevertheless, a desirable combination of actuation parameters, including strain, stress, energy density or power, is highly demanded for practically meaningful artificial muscle system, as well as reliable mechanical strength to endure a large external load in both actuated and relaxed states.

**Fig. 1:**
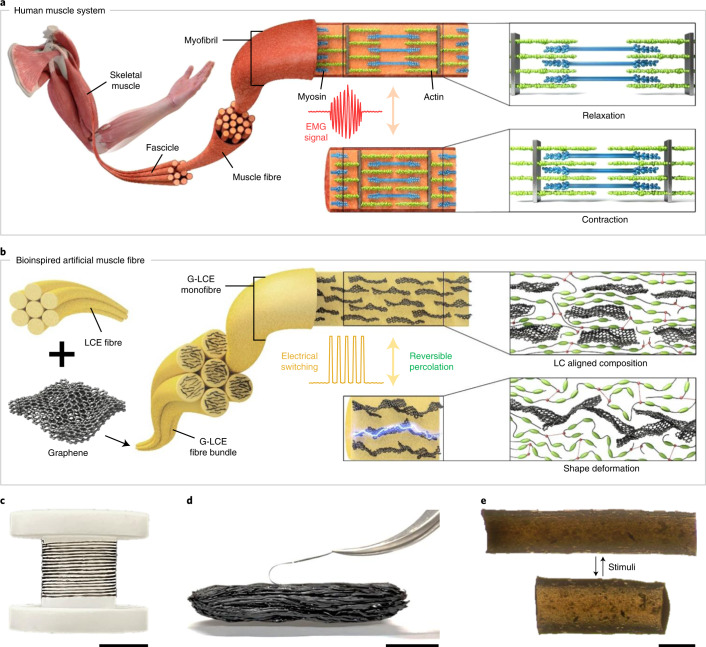
Human-muscle-like artificial muscle fibre. **a**, Structural organization and actuation mechanism of skeletal muscle consisting of myofibrils. **b**, Schematic of a G-LCF composite fibre for shape deformation mechanism with reversible percolation. **c**, Continuous G-LCF composite fibre wound over a reel. **d**, Animal-tissue-like 1,000 strand bundled structure of G-LCF artificial muscle fibres. **e**, Optical microscopy of relaxed and contracted states of G-LCF. Scale bars, 2 cm (**c**), 1 cm (**d**) and 200 μm (**e**).

As illustrated in Fig. 1a, myofibrils in human muscles linearly shorten by adjusting the spatial locations of constituent proteins called actin and myosin, whereas the length of the proteins remains constant^22^. The variation in action potential generates an electromyography (EMG) signal, which is commonly utilized for the detection of muscular operation in clinical/biomedical applications^23^. In our synthetic muscle fibres (Fig. 1b–e), liquid crystal elastomers (LCEs) are chosen as a large-strain reversible actuating matrix operated by the thermodynamic conformational transition of flexible polymer-chain segments across the nematic and isotropic (or paranematic) phases^24–33^. Unfortunately, the actuation power as well as response time of pure LCE actuators is limited by the intrinsic weak mechanical properties and retarded thermal relaxation. We address this challenge by incorporating graphene fillers with ultralarge shape anisotropy (average lateral size, <10 μm; layer stacking, <3), which are known to have strong mechanical properties and high thermal conductivity as well as outstanding photothermal conversion characteristics relying on wideband absorbance^34–36^.

It is well known that liquid crystalline (LC) moieties spontaneously self-assemble on graphene surfaces through strong π–π interactions and hydrogen bonding^37,38^. The precise control of the macroscopic alignment in one-dimensional fibre geometry along with the strong binding of LC moieties at the graphene surface enables an effective amplification of the mechanical properties, but the genuine large reversible actuation (up to 45%) is preserved. Graphene fillers also stimulate rapid actuation under near-infrared (NIR) irradiation, originating from the innate photothermal effect^39^. The resultant actuation performance attains work capacity and power density of 650 J kg^−1^ and 293 W kg^−1^, respectively, approximately 17 and 6 times of human muscle behaviours. Furthermore, many strands of soft muscle fibres can be easily assembled into hierarchical bundles, emulating sequentially gathered muscular tissues (that is, skeletal muscle, fascicle and muscle fibre) consisting of myofibrils (Fig. 1a and Supplementary Fig. 1). This offers a modular structural platform for living-body intimate complex actuating geometries along with the unlimited multiplication of working performances proportional to the number of fibres gathered (tested up to 1,000 strand bundling in this work).

More interestingly, the unique reversible assembly/disassembly of a highly anisotropic graphene filler network is preferentially induced in the fibre-axis direction on the substantially large contractive actuation/recovery in the one-dimensional fibre confinement, as easily monitored by electrical measurement (reversible ~10^4^-fold current change). Moreover, the reversible reconfiguration of percolation network overcomes the inherent mechanical weakening of actuator fibre in the contracted actuated state to ensure the mechanical endurance over the entire actuation cycle without interrupting the substantial dimensional change on actuation and relaxation. To the best of our knowledge, this is the first fibre-type soft actuator with thermodynamically driven reversible percolation of nanoscale filler network.

## Human-muscle-inspired single-fibre actuator and bundles

**Fig. 2:**
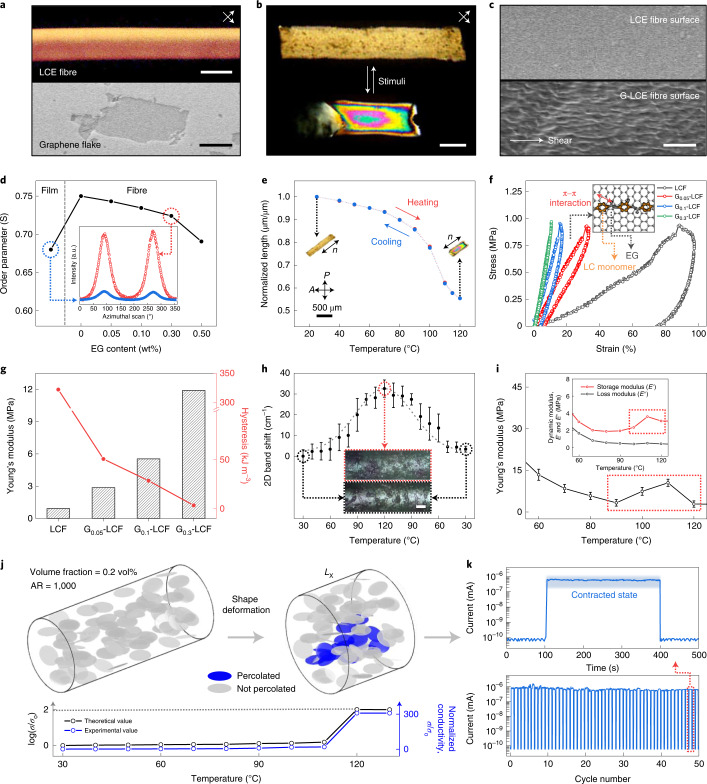
Highly aligned LCF and G-LCFs with reversible electrical percolation. **a**, POM image of LCF observed at 45° angles with respect to cross-polarizers (top) and SEM image of an EG flake (bottom). **b**, Shape deformation of G_0.3_-LCF under POM for relaxation and contraction. **c**, SEM image of LCF and G_0.3_-LCF surfaces. **d**, Orientational order parameters of LCF and G-LCFs from azimuthal scan profiles of 2D WAXS patterns. **e**, Reversible thermal actuation of highly aligned G_0.3_-LCF during stepwise steady-state heating and cooling cycle. **f**, Cyclic stress–strain curves for uniaxial tensile behaviours at 60 °C. The inset illustrates the π–π interaction between EG and LC mesogenic units. **g**, Young’s modulus and hysteresis level calculated from **f**. **h**, Raman 2D band shift against the contractive strain along the composite fibre as a function of temperature with the optical microscopy of relaxed and contracted states of G_0.3_-LCF. **i**, Variation in Young’s modulus and dynamic moduli of G_0.3_-LCF with temperature. **j**, Simulation for the reversible percolation of EG platelets under contractive actuation (volume fraction of 0.2% and aspect ratio (AR) of 1,000) along with theoretical and experimental values of electrical conductivity. **k**, Variation in electrical current of G_0.3_-LCF during reversible actuation over 50 cycles. Scale bars, 200 μm (top) and 2 μm (bottom) (**a**), 200 μm (**b**), 2 μm (**c**) and 50 μm (**h**). error bar=standard deviation for 10 measurements.

We optimized the direct melt spinning of LCE fibre (LCF) with or without graphene fillers (Fig. 2a, top, and Supplementary Information). Highly crystalline electrochemically exfoliated graphene (EG) fillers have been employed for achieving strong mechanical properties and high electrical conductivity (Fig. 2a, bottom). Homogeneously mixed precursors composed of EG fillers and LCE prepolymers were prepared by solution mixing in *N*,*N*-dimethylformamide (DMF), but the original thermodynamic characteristics of pure LCE are maintained (Supplementary Figs. 2 and 3). It is noteworthy that considering the large lateral size of graphene platelets used in this work (1–10 μm), more than hundreds of LC monomers are expected to multivalently assemble at each graphene filler surface with planar anchoring^37^. This intrinsic molecular affinity stabilizes the long-range reversible assembly and disassembly of the dispersed graphene fillers under a large dimensional change^40^.

Uniaxially aligned graphene LCE fibres (G-LCFs) exhibit reversible linear contractive dimensional change across the nematic–paranematic transition temperature (*T*_np_) of the LCE. Polarized optical microscopy (POM) initially verifies the nematic LC texture, which becomes paranematic under contraction (Fig. 2b). A well-aligned trace of EG fillers is observed at G-LCF surfaces in contrast to the featureless smooth surface of pure LCFs (Fig. 2c). Although EG fillers are well dispersed throughout the fibre volume, EG content over 0.5 wt% (G_0.5_-LCF) leads to considerable surface roughness arising from agglomeration. Orientational order parameter (*S*) of the LCE matrix shows a monotonic decrease with the EG loading from 0 wt% (LCF), 0.05 wt% (G_0.05_-LCF) and 0.10 wt% (G_0.1_-LCF) to 0.30 wt% (G_0.3_-LCF) and a sudden decline at 0.50 wt% (G_0.5_-LCF), arising from the agglomeration of EG (Fig. 2d). Taken together, G_0.3_-LCF was chosen as the optimal composition for a sufficiently high EG loading along with good LC alignment (Supplementary Fig. 4). It is noteworthy that the obtained *S* values in our fibre geometry are noticeably higher than those from the typical film-type LCE samples. The thermal actuation behaviour of G_0.3_-LCF (Fig. 2e, Supplementary Fig. 5 and Supplementary Video 2) exhibits a similar actuation temperature (*T*_a_) of ~100 °C with a negligible decrease in actuation strain compared with pure LCF (Supplementary Figs. 6 and 7).

The mechanical properties of our fibre actuators are significantly enhanced with the small composition of EG fillers beyond the typical requirement for practical applications (modulus, >10 MPa) not only in the original low-temperature relaxed state but also in the high-temperature contracted state (Supplementary Figs. 8–10). The resultant properties agree well with the theoretical values from a mean-field homogenization method^41^, verifying the stable dispersion of graphene fillers. As illustrated in Supplementary Fig. 11, our fibre actuators can also be easily assembled into large bundles by simple lateral contact among the aligned fibres, which is highly desirable for the multiplication of actuation strength and reliability. A brief thermal softening after bundling optimizes the van der Waals contact among the neighbouring fibres to constitute tight assembled structures from even more than 1,000 strands of single fibres. Apparently, the maximum tensile load for G_0.3_-LCF gradually increases up to 4.1 N with 100 strand bundling. The bundles of G_0.3_-LCF fibres show improved stiffness in the force–displacement curve, exhibiting 0.94 kN m^−1^ for the 100 strand bundle, but 0.06 kN m^−1^ for a single strand.

## Reversible percolation of graphene network in LCE matrix

Intimate intermolecular interactions among EG fillers and the LCE matrix could be evaluated by a uniaxial cyclic loading test with a dynamic mechanical analyser under a constant stress of 1 MPa, where elastic hysteresis loop curves were obtained after the removal of external force (Fig. 2f,g and Supplementary Fig. 12). The resilience nature of G-LCFs becomes more pronounced with EG loading, as evidenced by the dramatic decrease in hysteresis along with the rapid increase in Young’s modulus. The area of the enclosed hysteresis loop (measured under mild heating at 60 °C for contractibility) represents the typical energy-dissipative characteristics^42,43^ that decreases from 322.00 kJ m^−3^ (pure LCF) to 3.32 kJ m^−3^ (G_0.3_-LCF). This reconfirms that the minor composition of EG dominates the mechanical properties of G-LCFs. A significant decrease in the residual strain of G-LCFs was also observed after the complete relaxation of stress, compared with pure LCF, verifying the vigorous transformation from viscous to elastic characteristics with EG fillers (Supplementary Fig. 13). Significantly, a similar elastic response with minimal hysteresis is reproduced in the isotropic contracted states of G-LCFs, where pure LCE is generally known to show a high-energy-dissipative viscous character with severely weakened mechanical properties.

Raman spectroscopy is useful to directly investigate intermolecular interaction in our hybrid structure (Fig. 2h and Supplementary Fig. 14). G_0.3_-LCF reveals a redshift in the graphene two-dimensional (2D) band from the original pristine state, arising from the highly stretched graphene conformation within a well-aligned LCE matrix. On a contractive actuation, a favourable interaction among the EG fillers and LCE effectively delivers compressive stress to EG fillers, resulting in a reversible blueshift in the 2D band (phonon hardening)^44^. Noticeably, this tight intermolecular interaction brings about a reversible selective construction of the percolated graphene filler network within the composite structure only in the contracted state, as visualized by X-ray microscopy and scanning electron microscopy (SEM) for the internal structure and surface morphology, respectively. Non-destructive X-ray microscopy investigation on the EG filler configuration in the relaxed and contracted states of the fibre presents the assembly/disassembly of the filler network along the aligned direction of the fibre axis (Supplementary Fig. 15). The surface roughness of the fibre observed by SEM is also distinctive for pure LCF, relaxed G_0.3_-LCF and contracted G_0.3_-LCF with or without EG filler network generation within the LCE matrix (Supplementary Fig. 16). In addition, Young’s moduli obtained from the mechanical tensile tests of G_0.3_-LCFs at different temperatures exhibit an unexpected increase from 100 to 110 °C, as a result of the percolated network formation that predominates over the loss of LC ordering (Fig. 2i and Supplementary Fig. 17a). In this transition, the storage modulus of G_0.3_-LCF shows a pronounced increase with temperature, revealing a clear distinction from that of pure LCF (Supplementary Fig. 17b). The percolated network construction from the originally separated EG fillers obviously strengthens the elasticity of the heterostructure. It is noteworthy that a fully developed percolation network within a soft actuator matrix may disturb the effective dimensional change on actuation^45^. By contrast, our reversible percolation successfully addresses the inherent poor elasticity of LCE in the absence of LC ordering, effectively securing its innate large contractive actuation behaviour across the LC phase transition.

A numerical simulation was performed for the systematic understanding of reversible percolation behaviour (Fig. 2j and Methods). EG fillers were considered as monodisperse oblate rigid bodies for mathematical simplicity, whereas affine deformation was assumed for the position and orientation of EG fillers^45^. Initially, EG fillers are relatively well aligned and separated without forming a continuous network. Uniaxial contraction of 45% in fibre length (*L*_x_) brings about physical contacts among the fillers with rather randomized orientations, building up a percolated network. Normalized electrical conductivity measured across our fibre length exhibits a drastic increase over 120 °C, effectively matching with the simulation result. In the real-time measurement of reversible actuation under photothermal NIR manipulation, a significant variation in electrical current (maximum 10^4^-fold) was repeated over 50 cycles (Fig. 2k). Interestingly, a considerably larger variation in electrical current was detected in our fibre structures compared with the typical film samples with the same EG filler content (Supplementary Fig. 18). This could be successfully simulated by considering the preference for the in-plane orientation of planar EG platelets in film-type samples, as well as simple uniaxial alignment in the fibre samples. The percolated fraction of fillers is obviously higher in the fibre geometry on contraction. The electrical switching behaviour was further characterized at various EG contents and temperatures (Supplementary Figs. 19 and 20a). Although all the G-LCFs exhibit significant decreases in the linear electrical resistance at around 120 °C, G_0.3_-LCF attains the largest conductivity up to 5.1 × 10^−5^ S m^−1^ (Supplementary Fig. 20b), sufficient to turn on a candescent bulb at a voltage of 9 V (Supplementary Fig. 21).

## Rapid photothermal actuation with high actuation energy

**Fig. 3:**
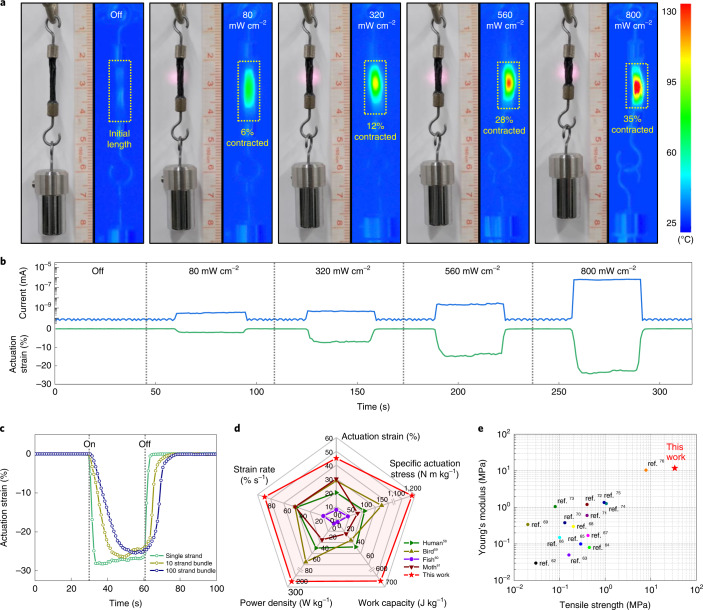
Rapid photothermal actuation of G-LCFs. **a**, Photoactuation and corresponding surface temperature variation in 100 strand G_0.3_-LCF bundle suspending 10 g weight subjected to NIR irradiation (*λ* = 808 nm) at room temperature. **b**, Actuation strain and concurrent electrical current change at the single-fibre level of G_0.3_-LCF induced by different NIR intensities under a constant stress (1.5 MPa). **c**, Contraction strains for different strands of G_0.3_-LCF in response to NIR irradiation (800 mW cm^-2^) under a constant stress (1.5 MPa). **d**, Comparison plot in terms of specific actuation stress, actuation strain, strain rate, work density and power density with natural biological muscles. **e**, Comparison of our actuator fibre with previous LCE-based actuators in terms of tensile strength and Young’s modulus in the contracted actuation states.

Graphene is an excellent photothermal converter with broadband absorbance, relying on the thermal dissipation mechanism via *sp*^2^-hybridized carbon lattice vibration^46^. The photothermal heating of EG fillers can induce the nematic–paranematic phase transition in the neighbouring LCE matrix within the thin fibre geometry, enabling a site-specific remote-controlled actuation. It is noteworthy that graphene has a distinctively higher photothermal conversion efficiency than other 2D materials, such as transition metal dichalcogenides or MXenes^47,48^. Figure 3a presents the photothermal actuation of a 100 strand bundle of G_0.3_-LCFs suspended with 10 g weight under NIR laser irradiation (*λ* = 808 nm). The surface temperature rapidly increased with the intensity of laser irradiation to reach 130 °C (that is, higher than *T*_np_) at 800 mW cm^−2^ (Supplementary Fig. 22), resulting in a contraction of up to 35%, exceeding the typical contraction rate of mammalian skeletal muscles (~20%). The photothermal mechanism enables a noticeably rapid actuation response under the on–off switching of NIR light (Supplementary Fig. 23). G_0.3_-LCF revealed the response time of 1.25 s and relaxation time of 0.30 s under 800 mW cm^−2^, approximately five times faster than conventional thermal heating. The response time could be further decreased down to 0.5 s with the NIR intensity of ~2,000 mW cm^−2^, beyond which thermal degradation occurred.

For the quantitative analysis of photothermal actuation, G-LCFs mounted on a tension clamp of the dynamic mechanical analyser with a constant strain of 3% were directly irradiated with NIR laser. Although all the G-LCF single fibres revealed rapid actuation (Supplementary Figs. 24 and 25), the actuation stress increased with EG content: 0.12 MPa for G_0.05_-LCF, 0.46 MPa for G_0.1_-LCF and 1.22 MPa for G_0.3_-LCF, respectively. A proportional relationship is also observed between the actuation stress and NIR intensity. This dependency of actuation stress on filler composition or light intensity is associated with the enhancement in photothermal effect as well as mechanical hardening of the LCE matrix with EG loading. Figure 3b presents the gradual increase in the actuation strain of G_0.3_-LCF proportional to NIR intensity under a preload of 1.5 MPa. This verifies the origin of photothermal conversion from EG fillers and the subsequent propagation of nematic–paranematic transition towards the neighbouring LCE matrix. Multicycle NIR tests revealed that the actuation stress and strain are relatively well retained over repeated cycles (Supplementary Figs. 26 and 27), even with the high intensity of IR laser for the largest actuator behaviour.

Photothermal actuation was also tested with fibre bundles (10 and 100 strands; Fig. 3c and Supplementary Figs. 1 and 28), which maintains the original large actuation strain against higher preload forces (applied stress was identically fixed as 1.5 MPa). The response time for full contraction, however, increases from 1.5 s (single strand) to 8.0 s (10 strands) and 15.0 s (100 strands) due to the screening of inner strands in a bundle from laser irradiation. Under a variation in the preload, the actuation strain of G_0.3_-LCF single strand gradually decreases above 1.5 MPa, whereas those of 10 and 100 strands considerably deform up to 3.0 MPa (Supplementary Figs. 29 and 30). Notably, the 100 strand bundle reveals a slightly lower maximum actuation strain compared with the 10 strand bundle, attributed to the non-uniform irradiation of NIR over many strands of fibres in the large bundle. Consequently, work capacities (density of G_0.3_-LCF was measured as 1,070 kg m^−3^) for single, 10 and 100 strands are measured to be 439, 650 and 522 J kg^−1^, respectively.

Figure 3d highlights the typical actuation parameters, including specific actuation strain, stress, work capacity, strain rate and power density of our artificial muscle, calculated with the corresponding response time, all of which are noticeably higher than those from typical natural muscles in living systems (Supplementary Fig. 31 and Supplementary Table 1). The actuation stress and energy values are on the same order with those of interpenetrating-polymer-network-based LCE actuators, the optimal interwoven macromolecular matrix with the highest values up to date (Supplementary Table 2). It is noteworthy that in addition to the strong and tough actuation behaviour itself, practical muscles inevitably require reliable mechanical strength in both actuated and relaxed states to endure large mechanical loads under powerful movements. Figure 3e demonstrates that our muscle fibre records the highest tensile strength and Young’s modulus particularly in the full contraction state among all the previous LCE-based actuator systems reported thus far, which is highly advantageous for realistic biomimetic actuation and locomotion as follows.

## Strong bundle structure and high-power soft robotics

**Fig. 4:**
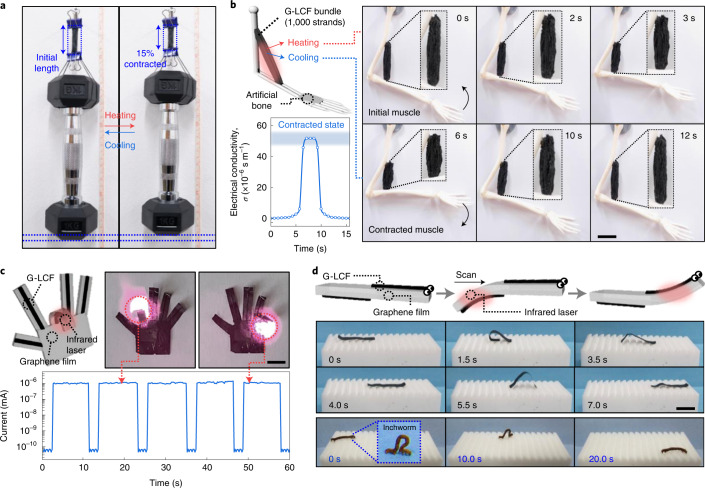
Demonstration of artificial-muscle-based customized actuators. **a**, A 1,000 strand bundle of G_0.3_-LCF lifting a 1 kg dumbbell via thermal actuation. **b**, Reversible actuation of animal-tissue-like artificial muscle bundle (1,000 strands) attached between an artificial human joint along with the corresponding electrical conductivity variation. **c**, Artificial finger operation under NIR remote control with electrical current variation. **d**, Biomimetic self-walking of all-carbon artificial worm driven by NIR manipulation. The natural movement of an alive inchworm for comparison. Scale bars, 2 cm (**b**) and 1 cm (**c** and **d**).

Our artificial muscles with flexible single-fibre geometry allow a large degree of freedom for the complex morphing of actuating bodies. Taking advantage of the strong mechanical properties and high-power actuation, heavy weight lifting is demonstrated with a 1,000 strand bundle of G_0.3_-LCFs (200 mg), where a 1 kg dumbbell (5,000 times heavier than the fibre bundle’s own weight) is successfully lifted (Fig. 4a and Supplementary Video 3). Without a reversibly percolated graphene filler network, it is difficult to endure such a heavy weight particularly in the contracted paranematic state, as it commonly leads to the sudden elongation or breakage of pure LCE actuators even after an energetic actuation. As shown in Fig. 4b, our animal-tissue-like bundle could be installed at the elbow joint of an artificial human arm and repeatedly actuated, as motivated from human bicep movement (Supplementary Fig. 32).

Palm and fingers in an artificial hand were fabricated by implementing single-fibril muscles to a customized graphene film (Fig. 4c and Supplementary Video 4). Each finger can be remotely controlled for the selective bending and straightening with a rapid operation time (<1 s) under NIR irradiation, along with the in situ monitoring of electrical conductivity. Last, an ultralight all-carbon artificial worm (0.88 mg) is demonstrated, which can self-crawl on a ratchet surface by photothermal manipulation (Fig. 4d). Two strands of G_0.3_-LCFs were attached at the opposite sides of a narrow graphene ribbon in the longitudinal direction. NIR manipulation induced an anisotropic motility consisting of sequential large bendings, originating from the photothermal actuation of G-LCFs at the ribbon surface (Supplementary Video 5), yielding the driving force to crawl in a programmed manner. A natural inchworm generates a looping gait similar to this artificial locomotion. Although a natural alive inchworm took ~20 s to travel across the 5-cm-wide substrate (2.5 mm s^−1^), our soft robot finished the same distance within 7 s (7.2 mm s^−1^). It is noteworthy that the crawling speed of our 15-mm-long artificial worm, to the best of our knowledge, is in the highest range among the previous soft crawlers based on various actuation mechanisms (Supplementary Table 3). Besides, our fibre actuator can be readily woven together with conventional cotton yarns to create versatile smart textiles (Supplementary Fig. 33). The directional deformation of a G-LCF-woven torn fabric as well as pore-size switchability was induced by NIR remote control (Supplementary Video 6).

## Conclusions

Synergistic material design exploiting molecular-level intimacy attains human-muscle-like actuator filaments and bundles, as well as realizes a desirable combination of actuation parameters required for practical utilization. Significantly, the accompanied reversible percolation of graphene fillers not only enables a facile monitoring of actuation behaviour but also selectively reinforces the mechanical performance under substantial contraction to ensure reliable biomimetic actuation and locomotion. This large degree of freedom in the tailoring of high-energy synthetic actuation modes opens up an intriguing platform widely applicable to smart clothing, reconfigurable Internet of Things devices, implantable muscular reinforcements, humanoid robotics and so on^49–52^.

## Methods

### Materials

1,4-Bis[4-(6-acryloyloxyhexyloxy)benzoyloxy]−2-methylbenzene (RM82) was obtained from Synthon Chemicals. Also, *n*-butylamine was purchased from Sigma-Aldrich. Irgacure-369 (I-369) was donated by BASF Corporation. Graphite foil was purchased from Alfa Aesar. Sodium sulfate (Na_2_SO_4_) was purchased from Junsei Chemical. All the materials were used without purification.

### Preparation of EG

Natural graphite foil (0.254 mm thick) was used as the anode, whereas a Pt wire was used as the cathode for the electrochemical exfoliation of graphite. An ionic solution of electrolyte was prepared by dissolving 1.42 g Na_2_SO_4_ in 100 ml H_2_O (0.1 M). A graphite foil was adhered to a copper wire and subsequently immersed in the ionic solution. The distance between the parallel positioned electrodes was fixed at 2 cm. The electrochemical exfoliation process was carried out by applying a.c. bias on the graphite electrode (from –10 to +10 V). After the completion of exfoliation, the product was collected by HVLP membrane filter with 0.45 µm pore size under vacuum filtration and washed several times with 0.1 M HCl solution to remove the ionic residues. After drying, the resultant EG was dispersed in DMF under mild sonication. Unwanted poorly exfoliated large graphite flakes were removed by centrifugation at 2,000 rpm for 30 min. All the electrochemical exfoliation experiments were performed at room temperature.

### Synthesis of LCO-based dope

In a 20 ml vial, RM82, *n*-butylamine and I-369 were introduced where the molar ratio of RM82 to *n*-butylamine was 1.01:1.00, and the amount of I-369 was 2.5 wt% of the total LC mixture^24,53^. The mixture was vigorously vortexed under heating for uniform mixing. Subsequently, the mixture underwent aza-Michael-addition-based step-growth polymerization (chain extension between diacrylate and amine) at the temperature of nematic phase (60–80 °C) for 24 h, resulting in step-growth oligomerization. The liquid crystalline oligomer (LCO) dope was kept at 5 °C to prevent additional reactions. For the EG-containing LCO dope, the desired volume of EG dispersion in DMF (1.15 mg ml^–1^) was added to a 20 ml vial containing LC mixture, and vortexed for 5 min. Afterwards, the EG–LC mixture was placed at 75 °C in a vacuum oven for 24 h, which allowed both oligomerization and DMF removal.

### Preparation of LCF and G-LCFs

The LCO dope with or without EG was transferred to an injection stainless steel syringe. The LCO dope was extruded through a nozzle (inner diameter, 400 μm), and collected onto a glass substrate moving laterally by homemade equipment. During extrusion, the dope was heated with a heating coil to a temperature of 15 °C below the nematic–paranematic transition temperature (*T*_np_). The end of the nozzle spinneret was positioned 1 mm away from the collector. The extruded fibres were quenched at room temperature and subsequently exposed to ultraviolet light (OmniCure S1500, *λ* = 365 nm and 30 mW cm^−2^) for 30 min for photopolymerization, producing LCF or G-LCFs. Then, the products were immersed in water to strip off from the glass substrate, and recollected with a Teflon reel.

### Mean-field homogenization method

The Mori–Tanaka method, one of the widely used mean-field homogenization schemes, was applied to predict the effective Young’s modulus of G-LCFs with respect to the EG flake content. Our theoretical prediction was compared with the experimental results. Because the Mori–Tanaka method assumes that particles are very well dispersed and that the particle–matrix interface is very strongly bonded, the mismatch between theoretical prediction and experimental measurements indicates that either EG flakes are agglomerated or the interface between the EG flake and matrix is very weak. Because of the strong π–π interaction, the interfacial strength would be strong for the composite in the present study, and thus, the mismatch probably originates from EG agglomerations. Since it is mathematically convenient to apply the homogenization methods for ellipsoidal particles, the EG flake was modelled as an oblate spheroid with an aspect ratio of 1,500. EG flakes were assumed to be aligned by the manufacturing process. Since homogenization takes account of the volume fraction instead of weight fraction, note that 0.3 wt% of weight fraction was roughly converted to 0.45 vol% of volume fraction. The Young’s modulus of LCE was set to be 12.8 MPa, which is the initial slope obtained by experiment. The Young’s modulus of the EG flake was set to be 1 TPa, and Poisson’s ratios for both LCE and EG flake were set to be 0.3 for simplicity. Note that the actual properties of EG are different for the in-plane and out-of-plane directions. The effective properties of the composite for the Mori–Tanaka method were expressed as follows:

$${L}_{{\rm{eff}}}=\left({c}_{0}{L}_{0}+{c}_{1}{L}_{1}:T\right):{\left({c}_{0}I+{c}_{1}T\right)}^{-1},$$

$$T={\left(I+S:{L}_{0}^{-1}:({L}_{1}-{L}_{0})\right)}^{-1},$$

where *c*_0_ and *c*_1_ are the volume fractions of the matrix and particles, respectively, and *I* is the fourth-order identity tensor. *L*_0_, *L*_1_ and *L*_eff_ are the stiffness tensors of the matrix, particle and composite, respectively. Here *S* is the Eshelby tensor—a function of the shape of the particle and material properties of the matrix. The analytical expression of the Eshelby tensor for the isotropic matrix with an oblate spheroid particle can be found in previous research^54–56^.

### Simulation for modelling percolation network formation on deformation

EG platelets were assumed to be an oblate rigid body due to their high modulus as well as for mathematical simplicity. The aspect ratio was set to 1,000 and the volume fraction was 0.45 vol% (that is, 0.3 wt%). Also, an affine transformation was assumed for the position and orientation of the EG platelets on tensile deformation. When compressive strain is applied in the *x* direction, the centre and orientation of the EG platelets were represented as follows:

$$\left[{x}_{{\rm{c}}},{y}_{{\rm{c}}},{z}_{{\rm{c}}}\right]=\left[\left(1+\varepsilon \right){x}_{c0},\frac{{y}_{c0}}{\sqrt{1+\varepsilon }},\frac{{z}_{c0}}{\sqrt{1+\varepsilon }}\right],$$

$$\theta {=\tan }^{-1}\left[\frac{{\rm{\tan }}{\theta }_{0}}{\left(1+\varepsilon \right)\sqrt{1+\varepsilon }}\right],\phi {=\tan }^{-1}\left[{\rm{\tan }}{\phi }_{0}\frac{{\rm{\sin }}{\theta }_{0}}{{\rm{\sin }}\theta }\right],$$

where [*x*_c_, *y*_c_, *z*_c_] is the centre position of each graphene platelet, *θ* (range, from –π/2 to π/2) is the angle between the projection of the semi-major axis on the *x*–*y* plane and *x* axis, *ϕ* (range, from 0 to π) is the angle between the semi-major axis and *z* axis, subscript 0 means the initial state and each initial value is randomly chosen. A connectivity analysis was conducted to verify the percolation of EG platelets. First, the distance between two graphene platelets was calculated; then, if the distance is smaller than the cutoff contact distance, two platelets were considered to be in contact. Second, based on the obtained contact information, the depth-first search algorithm was used to identify a cluster of EG platelets. In the depth-first search algorithm, we start at an arbitrary node and explore along each branch as far as possible. If the exploration is no longer available, the algorithm explores backwards on the same path to find other nodes to traverse. Finally, a cluster of EG platelets is obtained and we could determine whether the EG platelets form percolated networks or not. Additionally, to obtain a rough conductivity value based on the assumption that the current in the graphene platelet flows in the shortest direction and the resistance is only proportional to the distance, Kirchhoff’s current law was applied to calculate the theoretical conductivity of the percolated graphene platelets. For the simulation of structure-dependent percolation fraction, we assumed the initial polar and azimuthal angles of the platelets. The polar angle (*θ*) is allocated a value near π/2 in both film and fibre structures to be aligned to the *x*_1_ axis. The azimuthal angle (*ϕ*) for the film structure has a value near 0 or π; therefore, it tends to be parallel to the *x*_1_–*x*_3_ plane. On the other hand, the azimuthal angle has a fully random angle in the fibre structure. Next, the percolation analysis was performed after the compression process with affine transformation. To obtain a reliable result, 30 simulations were performed under each structure.

### Characterization techniques

A polarized optical microscope (Nikon Eclipse LV100N POL) with a heating stage (Linkam LTS420) was used to characterize the alignment, phase transition and thermal actuation of LCFs and G-LCFs employing ToupView software. Thermal actuation was analysed by observing the changes in length and width of the fibres during heating and cooling under POM floating on silicone oil. Here *T*_np_ was determined by differential scanning calorimetry (TA Instruments Q20) under a nitrogen atmosphere. All the samples were heated to 150 °C, then cooled to −50 °C and reheated to 150 °C at the rate of 10 °C min^–1^. The morphology of the EG flake and cross-section of fibres were observed by a scanning electron microscope (Hitachi S-4800). The thickness of the EG flake was analysed with atomic force microscopy measurement using Innova (Bruker) under the tapping mode. The chemical composition of EG was measured with X-ray photoelectron spectroscopy (Thermo VG Scientific, Sigma Probe) analysed by Avantage software. The Raman spectrum was obtained by ARAMIS (Horiba Jobin Yvon) with 514 and 785 nm excitation laser. For the evolution of graphene 2D peak shift, ten spectra were obtained at each temperature to yield an average 2D band wavelength. Fourier-transform infrared measurements were performed with IFS 66v/s and HYPERION 3000 (Bruker Optics). Wide-angle X-ray scattering (WAXS) and X-ray diffraction measurements were carried out by D/MAX-2500 (Rigaku) using a beam with Cu Kα wavelength. Samples for WAXS measurements were prepared by aligning the fibres within rectangle homemade frames. The orientation order parameter of the fibres was calculated as (180° − *H*°) × 100/180°, where *H*° is the half-width of the intensity distribution on the azimuthally scanned peaks. Three-dimensional mapping images of the EG filler configuration during shape deformation were obtained by an X-ray microscope (Zeiss Xradia 520 Versa). Each image was taken by using a ×20 objective lens with 25 s exposure time under 60 kV and 5 W, resulting in a 0.7 μm resolution after fixing the relaxed and contracted states of the samples. All the electrical measurements were conducted using a semiconductor parameter analyser (Keithley 4200-SCS). During device operation, voltage was applied at the top electrode, whereas the bottom electrode was kept grounded. Five samples were examined for the average electrical conductivity value at each condition. A thermal imaging camera (FLIR A655sc) was used to observe the surface temperature of the composite. The response and relaxation time was acquired by analysing the frames from a high-speed camera as the time after infrared laser irradiation/removal to reach the length shrinkage/relaxation of 10%. An average value from ten measurements were obtained. The mechanical properties and photothermal actuation were characterized by a dynamic mechanical analyser (DMA Q850, TA Instruments) using a tension film clamp. For stress–strain curves, the samples were equilibrated at 30 °C under a preload of 0.01 N, and the force was increased at the rate of 0.2 N min^–1^. The tensile loading–unloading test was performed at different temperatures under a constant stress of 1 MPa with a preload of 0.001 N. The force was increased at the rate of 0.001 N min^–1^. During temperature sweep measurements, the sample was heated from 30 to 150 °C with a 10 °C increment under a constant frequency of 1 Hz with a preload of 0.001 N. All the values of the mechanical properties, including tensile strength, strain, toughness and stiffness, were averaged from more than five measurements. Photothermal actuation was carried out by irradiating an NIR laser (MDL-3) of 808 nm wavelength for five different fibre samples for each measurement condition. The photoactuation stress was measured by subjecting the samples to NIR irradiation under a constant strain of 3% (refs. ^57–94^).

## Online content

Any methods, additional references, Nature Research reporting summaries, source data, extended data, supplementary information, acknowledgements, peer review information; details of author contributions and competing interests; and statements of data and code availability are available at 10.1038/s41565-022-01220-2.

## Supplementary information

Supplementary Information Supplementary Figs. 1–40, Tables 1–3 and text.

Supplementary Video 1 Direct melt spinning of LCE-based fibre with homemade equipment.

Supplementary Video 2 Reversible shape deformation of G0.3-LCF under heat (×10).

Supplementary Video 3 High-power actuation with 1,000 strand bundle of G0.3-LCFs lifting a 1 kg dumbbell (×10).

Supplementary Video 4 Bending motion of all-carbon artificial hand under NIR irradiation.

Supplementary Video 5 Movement modulation mechanism of an all-carbon artificial worm (×0.25).

Supplementary Video 6 Directional deformation of G0.3-LCF-woven torn fabric under NIR irradiation.

## Data Availability

The data supporting the findings of this study are available within the Article and its Supplementary Information or from the corresponding author on request.
