# Supplementary material for: Human-muscle-inspired single fibre actuator with reversible percolation
Source: Nat Nanotechnol. 2022 Oct 27;17(11):1198–205. doi: 10.1038/s41565-022-01220-2 (PMC9646516; doi:10.1038/s41565-022-01220-2)
Supplement: Supplementary file 1 — Supplementary Figs. 1–40, Tables 1–3 and text. [file 41565_2022_1220_MOESM1_ESM.pdf]

nature nanotechnology

Article https://doi.org/10.1038/s41565-022-01220-2

Human-muscle-inspired single fibre actuator with reversible percolation

In the format provided by the authors and unedited

Supplementary information

Human muscle inspired single fiber actuator with reversible percolation

In Ho Kim[1,2], Subi Choi[4], Jieun Lee[4], Jiyoung Jung[5], Jinwook Yeo[5], Jun Tae Kim[1,2], Seunghwa Ryu[5], Suk-kyun Ahn[4], Jiheong Kang[1], Philippe Poulin[6], and Sang Ouk Kim[1,2,3]*

[1] Department of Materials Science and Engineering, Korea Advanced Institute of Science and Technology (KAIST), Daejeon 34141, Republic of Korea.

[2] National Creative Research Initiative Center for Multi-dimensional Directed Nanoscale Assembly, Korea Advanced Institute of Science and Technology (KAIST), Daejeon 34141, Republic of Korea.

[3] Materials Creation, Co., LTD. Donggu Building 3F, 15, Teheran-ro 86-gil, Gangnam-gu, Seoul 06179, Republic of Korea.

[4] Department of Polymer Science and Engineering, Pusan National University, Busan, Korea 46241, Republic of Korea.

[5] Department of Mechanical Engineering, Korea Advanced Institute of Science and Technology (KAIST), Daejeon 34141, Republic of Korea.

[6] Université de Bordeaux, CNRS, Centre de Recherche Paul Pascal, UMR5031, 33600 Pessac, France.

*To whom correspondence should be addressed:

E-mail: sangouk.kim@kaist.ac.kr

Supplementary text

Optimization of highly aligned LCFs

LCFs were designed by two-step process via direct melt-spinning, as schematically described in Supplementary Fig. 34a (see Supplementary Fig. 35 for the home-made equipment). First, LCO dope was extruded through the nozzle onto glass plate collector at 15 °C below from T_{np} (above 90 °C for all dopes) using heating coil, (Supplementary Video 1) as higher temperature (in isotropic phase) degraded the alignment of LC, while a lower temperature led to the solidification without fluidity. The mesogenic units with random direction in LCO dope were aligned into monodomain along the fiber axis under the external shear. Subsequently, cross-linked network of uniaxial alignment within LCFs was fixed via UV photopolymerization of diacrylate. This continuous melt-spinning process with home-made equipment renders continuous scalable fabrication of highly aligned elastomeric fiber as shown in Supplementary Fig. 34b. Supplementary Fig. 36 exhibits the evolution of alignment state for the as-spun fibers summarized with respect to the fiber draw ratio under various spinning parameters. The draw ratio was controllable by the speed of collector stage movement against the fiber extrusion rate. The gap distance between spinneret and collector plate (d) was kept constantly at 1 mm for the desirable alignment of mesogenic units. Draw ratio below 2 led to the accumulated fiber morphology at substrate surface. Fibers with the draw ratio over 2 showed optical birefringence under the rotatable crossed polarizers perpendicular to the direction of the light propagation verifying the preferential alignment of LC oligomers along the fiber axis (Supplementary Fig. 37a). While the birefringence becomes stronger along with draw ratio, discontinuous feature appeared above the draw ratio of 30. In the draw ratio of 10 to 30 significant thinning of fiber diameter was observed,

which is undesired for the practical treatment and actuating applications. Taken together, an optimal condition was established at the draw ratio of 8 to 9 for the monodomain LC alignment state of LCFs with sufficiently large fiber diameter of ~203 μm (Supplementary Fig. 37b).

Determination of molecular weight of LC oligomer

Mixture of nematic diacrylate monomer with chemical structure as shown in Supplementary Fig. 38a. The three peaks in the ^1H NMR spectrum (peak b, c, d in Supplementary Fig. 38b) appearing at 5.85-6.45 ppm correspond to six protons in the diacrylate end groups of the LCO. The integration value of these peaks was used to calibrate other peaks and set as six. The peak at 8.15 ppm (peak a) correspond to the four aromatic protons in the LCO with integration value of 71.31. The number of repeating unit (DP) was calculated by (71.31 / 4) – 1 = 16.8, and the M_n was calculated by DP × M_n of repeating unit (16.8 × 746 g mol^{-1}) + M_n of end-group (672 g mol^{-1}) = ~13200 g mol^{-1}.

Chemical analysis of EG

Electrically exfoliated graphene was prepared from graphite foil two electrode systems as shown in Supplementary Fig. 39a. SEM and AFM characterizations identified the average flake size of a 3.3 μm and thickness of 2.01 nm, respectively (Supplementary Fig. 39b to d). High-resolution of C 1s spectra of EG by XPS detected the C=C bonding as the major peak at 284.5 eV that corresponds to sp^2 hybridized carbons from the basal graphitic plane together with the additional peaks at 285.5 eV, 286.5 eV, 287.9 eV, and 288.9 eV assigned to C-C, C-O, C=O, and O-C=O bonding, respectively (Supplementary Fig. 40a)[57]. Carbon to oxygen (C/O) ratio within the C 1s peak from XPS data was calculated to be ~7, considerably higher than those of graphene oxide

(GO) and reduced graphene oxide (rGO)[58]. Further chemical analysis was investigated by Raman spectrum displaying an intense D peak at 1350 cm^{-1} and G peak at 1590 cm^{-1}, respectively (Supplementary Fig. 40b)[59]. EG shows the D/G ratio of 0.96 verifying the low degree of defects. A clear 2D peak at 2710 cm^{-1} associated with the presence of disorders was also detected[60]. The intensity ratio of 2D/G, which is relevant to the graphitization degree in carbon structure was obtained to be 0.26 for our EG, further suggesting the high quality compared to typical rGO. Attenuated total reflection Fourier transform infrared (ATR-FTIR) spectra of EG dispersions proves the highly exfoliated state with the presence of peaks for C-O-C stretching at 1050 cm^{-1}, C-OH stretching at 1365 cm^{-1}, and C=O stretching derived from carbonyl and carboxylic groups at 1720 cm^{-1} with skeletal vibration of graphitic domains at 1610 cm^{-1} as shown in Supplementary Fig. 40c[61].

Detailed mechanical properties characterization of LCF and G-LCFs

Stress-strain curves obtained from tensile tests exhibit semi-soft elastic plateaus originated from the innate ductility of LCE matrix, accompanied by the noticeable strengthening by EG loading. The tensile strength is dramatically enhanced with EG fillers (up to 15.1-folds for $G_{0.3}$-LCF), while preserving the maximum elongation below 200% for a well-aligned LC configuration. The increase of Young's modulus from 12.8 (pure LCF) to 80.6 MPa ($G_{0.3}$-LCF) shows a good agreement with the theoretically predicted values but significant deviation starts to occur above 0.75 vol% ($G_{0.5}$-LCF) due to the segregation of EG flakes. Toughness also proportionally increases with the EG content from 2.4 (pure LCF) to 22.2 MJ m^{-3} ($G_{0.3}$-LCF).

Comparative specification and performances of LCF and G-LCFs

Systematically optimized single LCE fibers show a regular diameter of 203 μm with or without graphene fillers and revealed light density below 1070 kg m^{-3}. Light driven actuation behaviors estimated by DMA (Q850) reveal ultrafast response time compared to the thermal-driven ones. In addition, these artificial muscles fibers with high contraction rate (45%) can be readily assembled into bundled structures. Correlated performances of the as-prepared artificial muscles such as work capacities and power densities calculated by considering above characteristics were examined in the single strand or fiber bundle level, as summarized in Supplementary Table 1. Overall, substantial enhancements of actuation performances were realized by homogeneous intercalation of graphene fillers in LCE network. Our genuine light-weight artificial muscle fibers showed outstanding actuation stress and work capacity arising from the enhancement of stiffness, as summarized in Supplementary Table 2. It is also noteworthy that our artificial muscle fiber system exceeded the most of previous LCE based actuators, specifically exhibiting the work capacity and power density approximately 3.5 and 17 times higher than natural human muscles. In addition, ultralight all-carbon artificial worm consisting of our high-power composite fibers also showed a superior crawling speed compared to the natural inchworms and attained one of the fastest values among the previous artificial soft crawlers based on diverse strategies of actuation as shown in Supplementary Table 3.

Supplementary figures

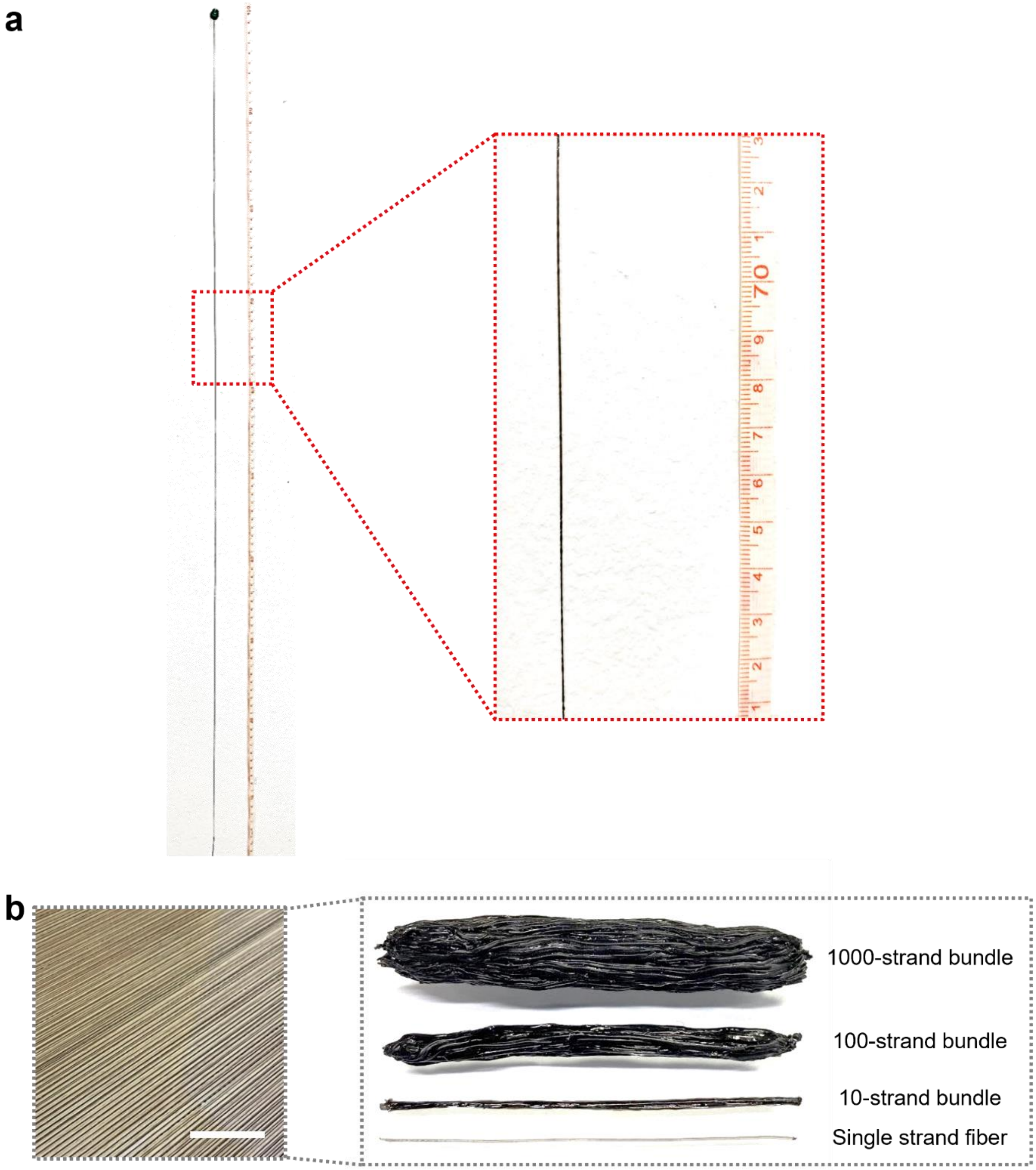

Supplementary Fig. 1 | a, Photograph of meter-long continuous single strand G-LCF. **b,** Single level G-LCFs deposited on the fiber collector surface and gathered bundle level of G-LCFs with different number of strands. Scale bar, 5 mm.

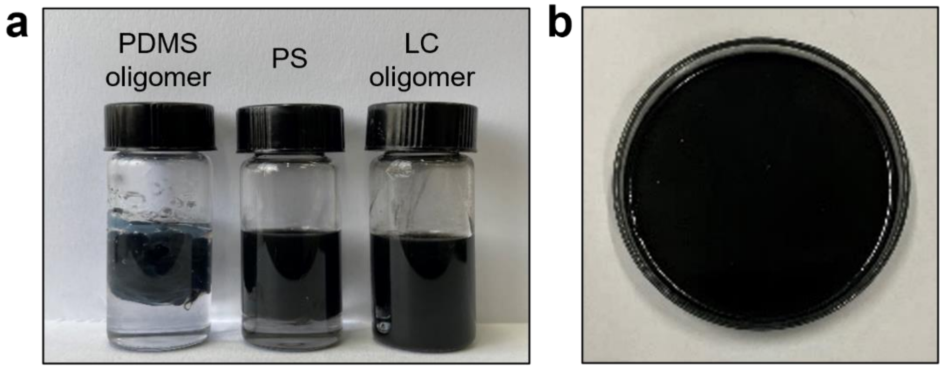

Supplementary Fig. 2 | a, Dispersion test of EG flakes in DMF with additional polydimethylsiloxane (PDMS) oligomer, polystyrene (PS), and LC oligomer in similar molecular weights for 3 weeks. **b,** Homogeneous EG-LCO hybrid dope with 0.3 wt% EG.

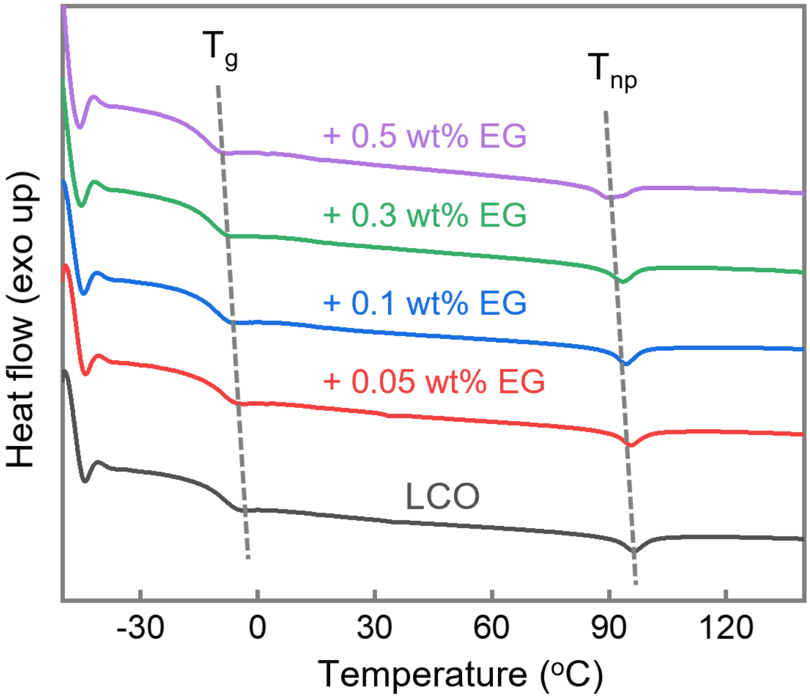

Supplementary Fig. 3 | DSC thermograms of LCO and LCOs with different EG loading levels upon second heating cycle.

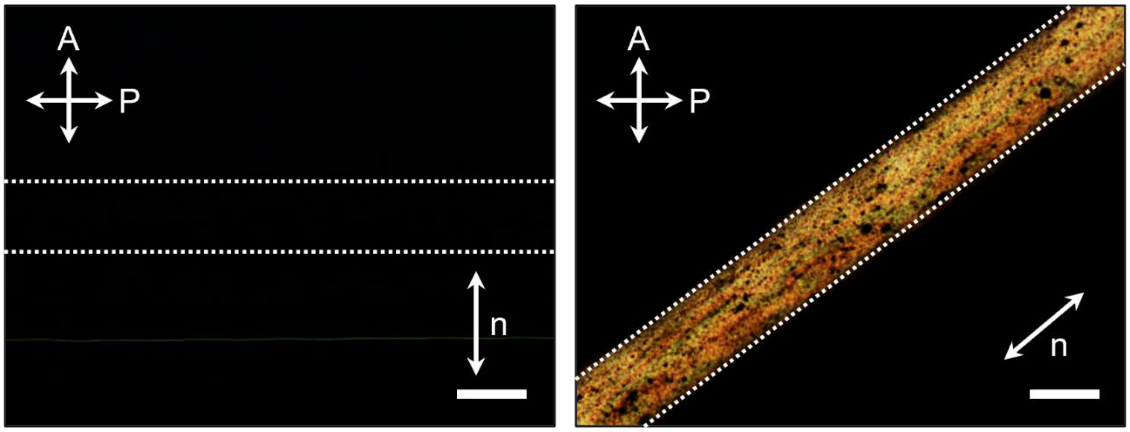

**Supplementary Fig. 4 | ** POM images of highly aligned $G_{0.3}$-LCF with different orientations of crossed polarizers. Scale bar, 200 μm.

[1st cycle]

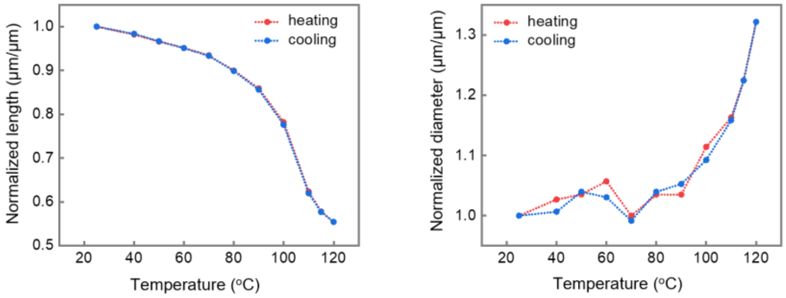

[2nd cycle]

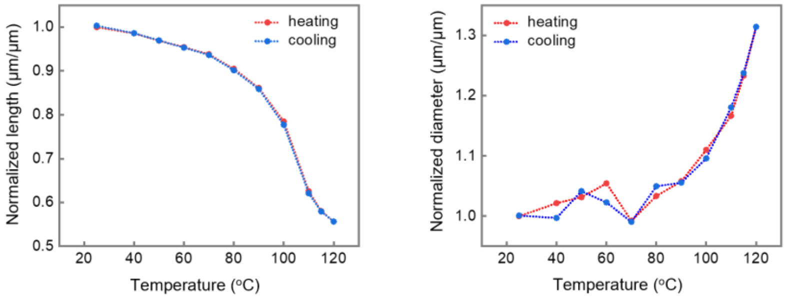

[3rd cycle]

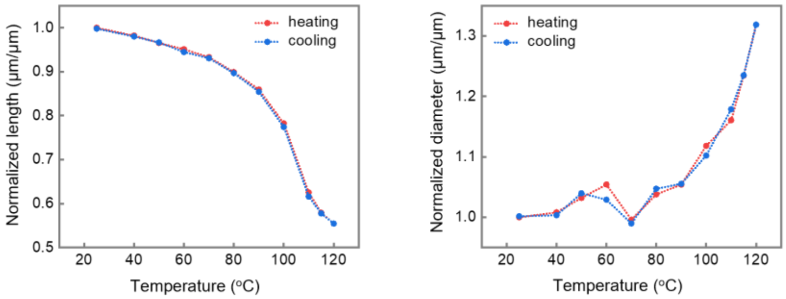

[4th cycle]

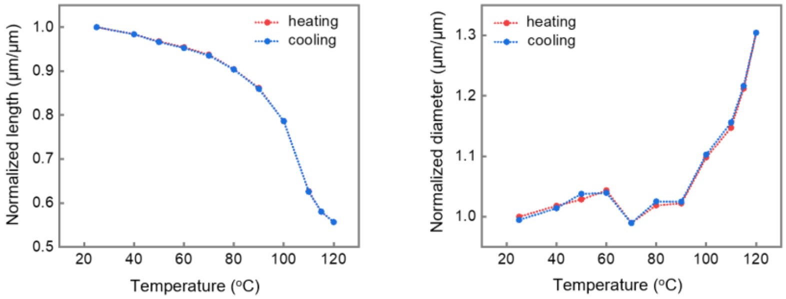

[5th cycle]

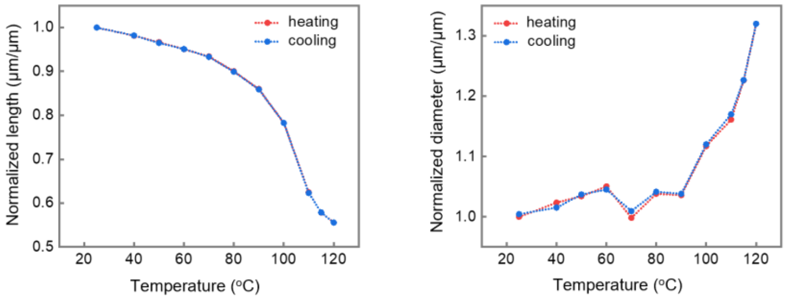

**Supplementary Fig. 5 | ** Reversible actuation of $G_{0.3}$-LCF in terms of the changes in length (left) and diameter (right) during 5 times of stepwise steady-state heating/cooling cycles.

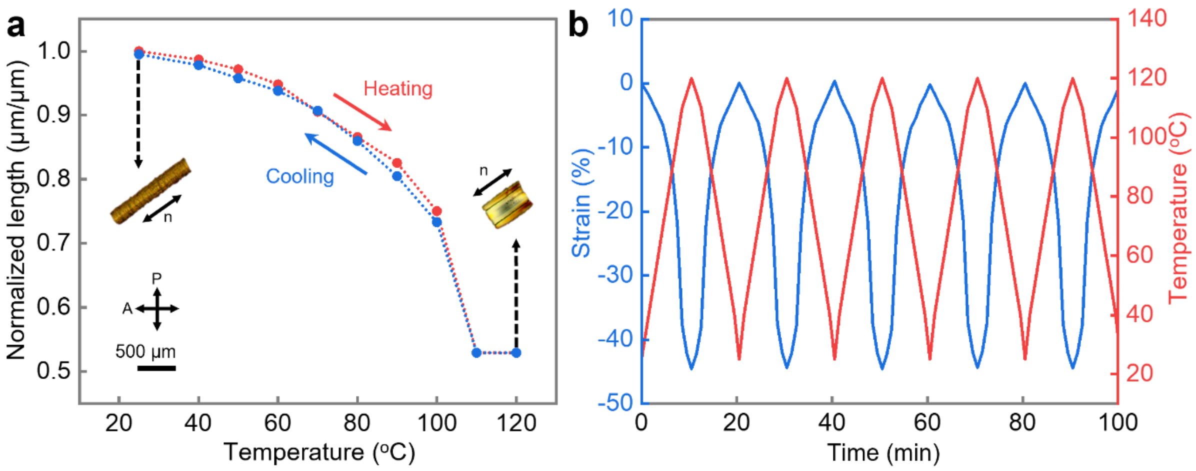

Supplementary Fig. 6 | a, Reversible thermal actuation of LCF in terms of changes in length and during heating and cooling. **b,** Reversible actuation during five cycles of temperature control.

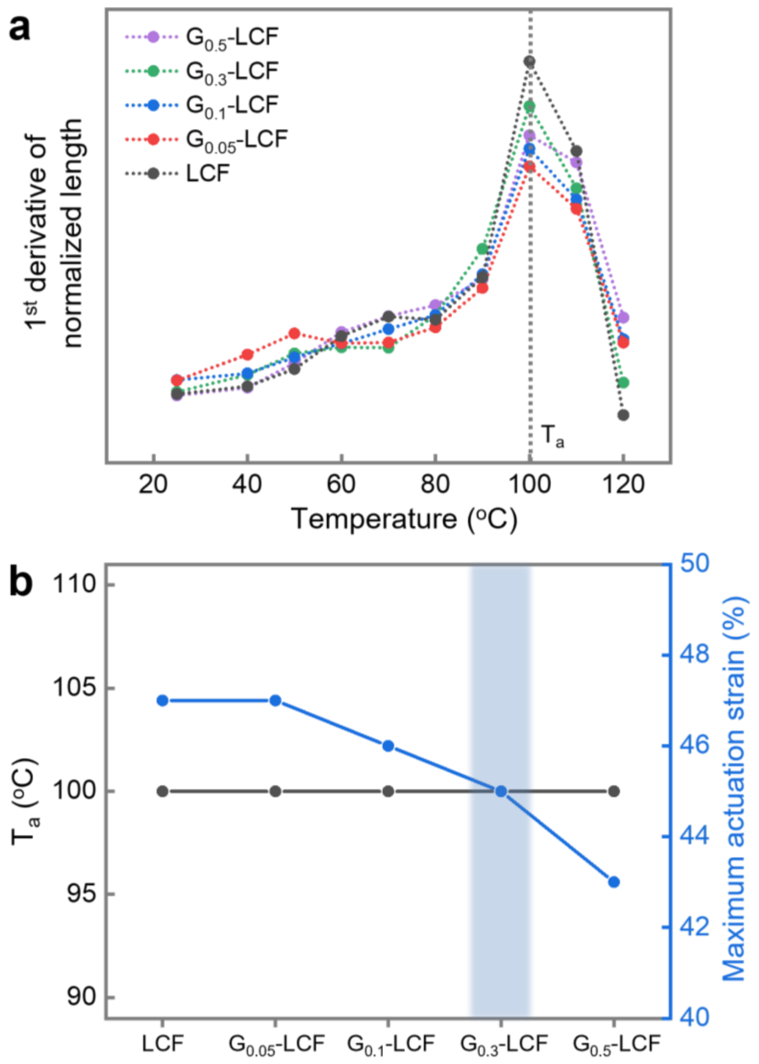

Supplementary Fig. 7 | a, The 1st derivatives of the normalized lengths of actuator fiber as a function of temperature. **b,** Evolution of T_a and maximum actuation strain of LCF and G-LCFs.

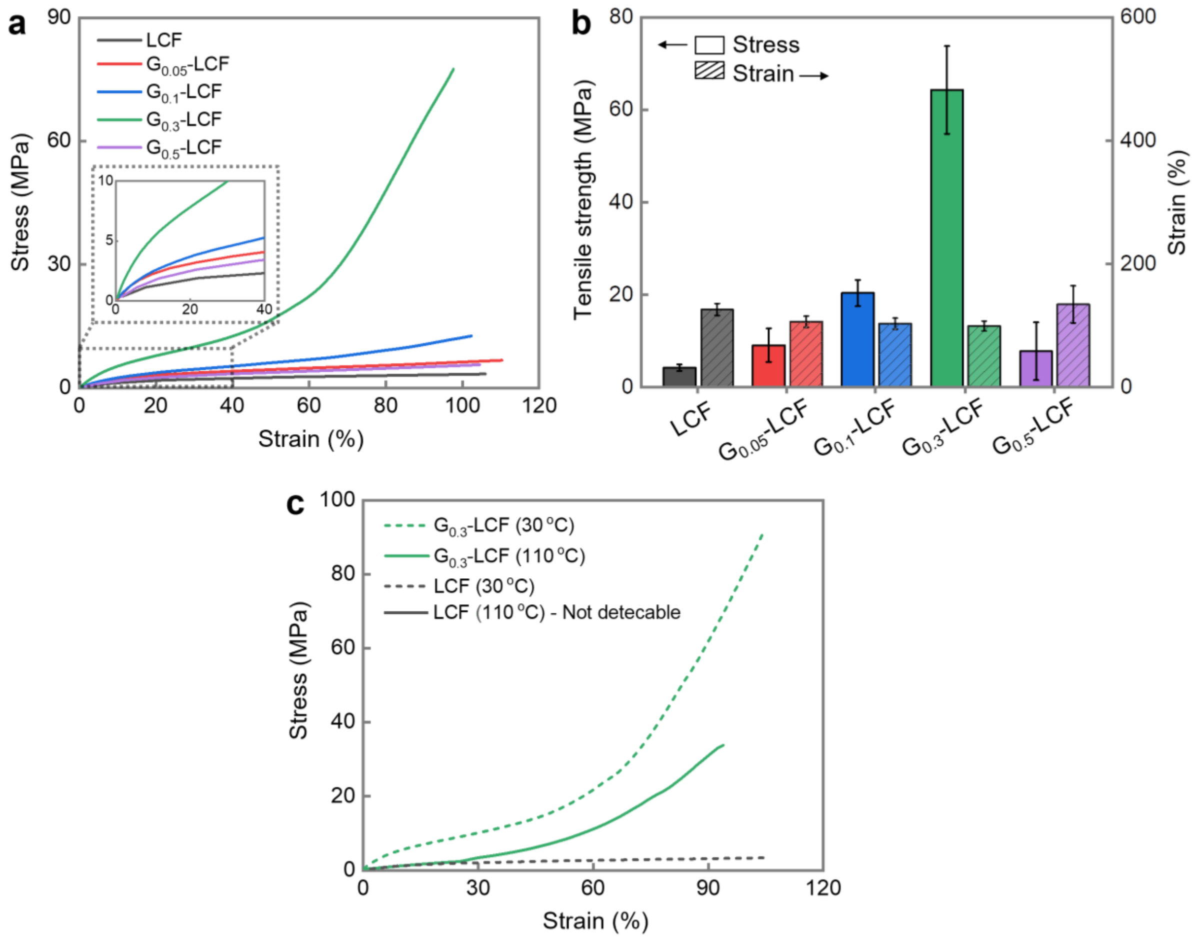

Supplementary Fig. 8 | a, Stress-strain curves for single fiber level LCF and G-LCFs at 30 ºC. **b,** Tensile stress and strain at break for LCF and G-LCFs. **c,** Stress-strain curves for LCF and $G_{0.3}$-LCF at original low temperature relaxed state and high temperature fully contracted state.

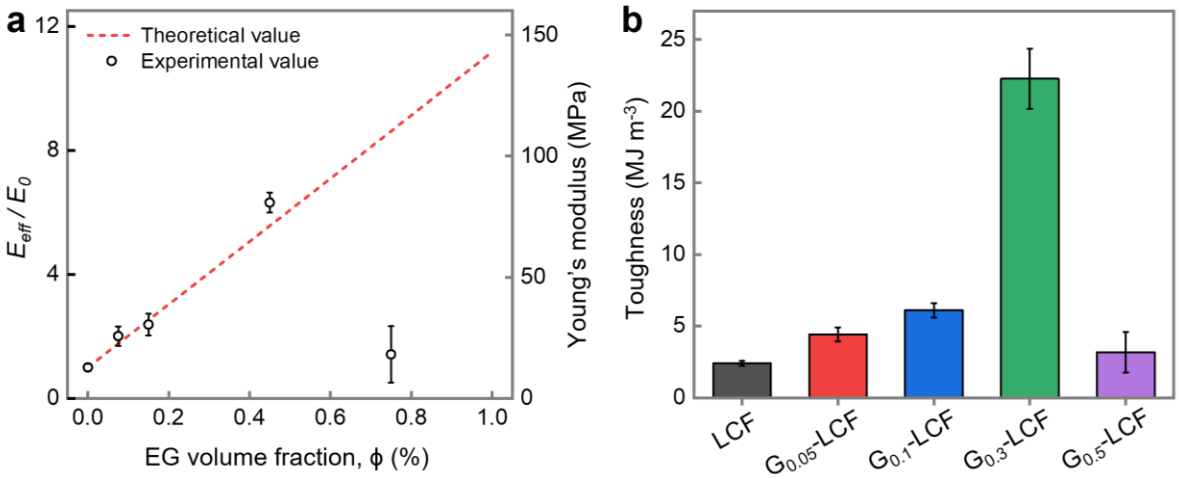

Supplementary Fig. 9 | a, Young's modulus of G-LCFs with theoretical prediction by a mean-field homogenization method (each point of volume fraction of 0.075, 0.15, 0.45, and 0.75 corresponds to 0.05, 0.1, 0.3, and 0.5 wt%, respectively). **b,** Toughness of single fiber level LCF and G-LCFs calculated from stress-strain curves.

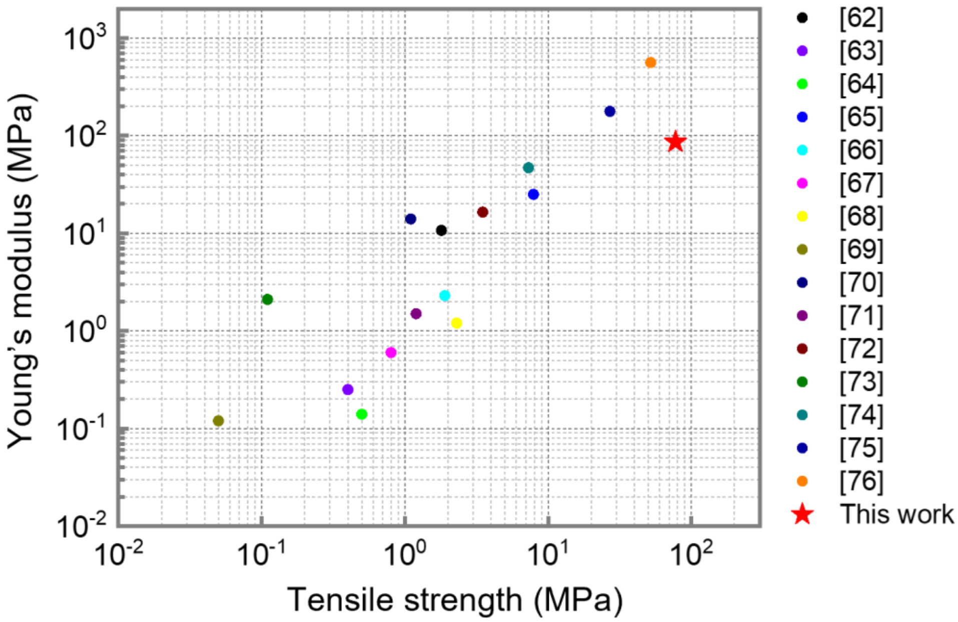

**Supplementary Fig. 10 | ** Comparison of our actuator fiber with previous LCE base actuators in terms of tensile strength and Young's modulus under ambient temperature.

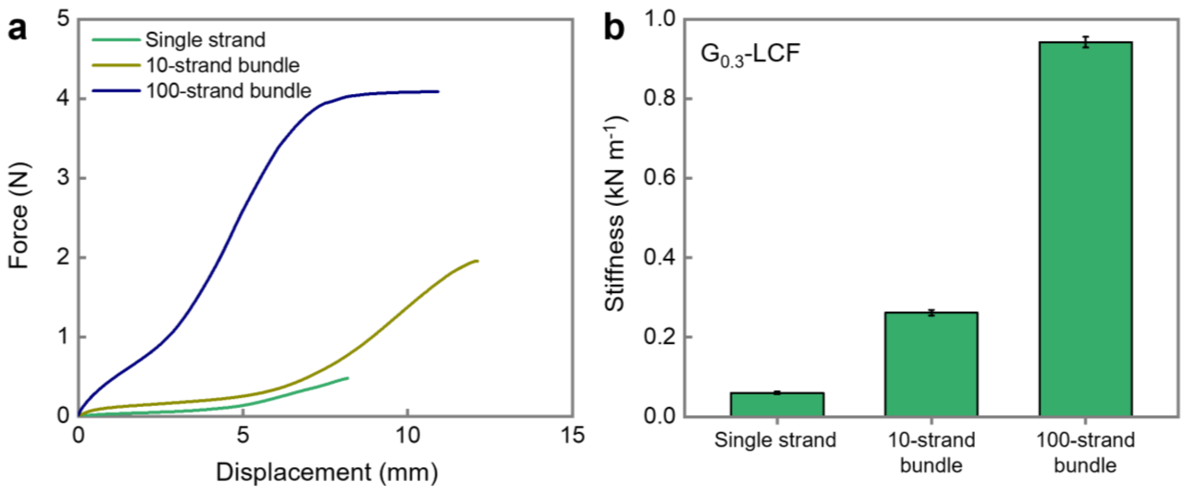

Supplementary Fig. 11 | a, Force-displacement curves of $G_{0.3}$-LCF with different number of strands at 30 ºC. **b,** Stiffness of single and bundle level of $G_{0.3}$-LCFs from force-displacement curves.

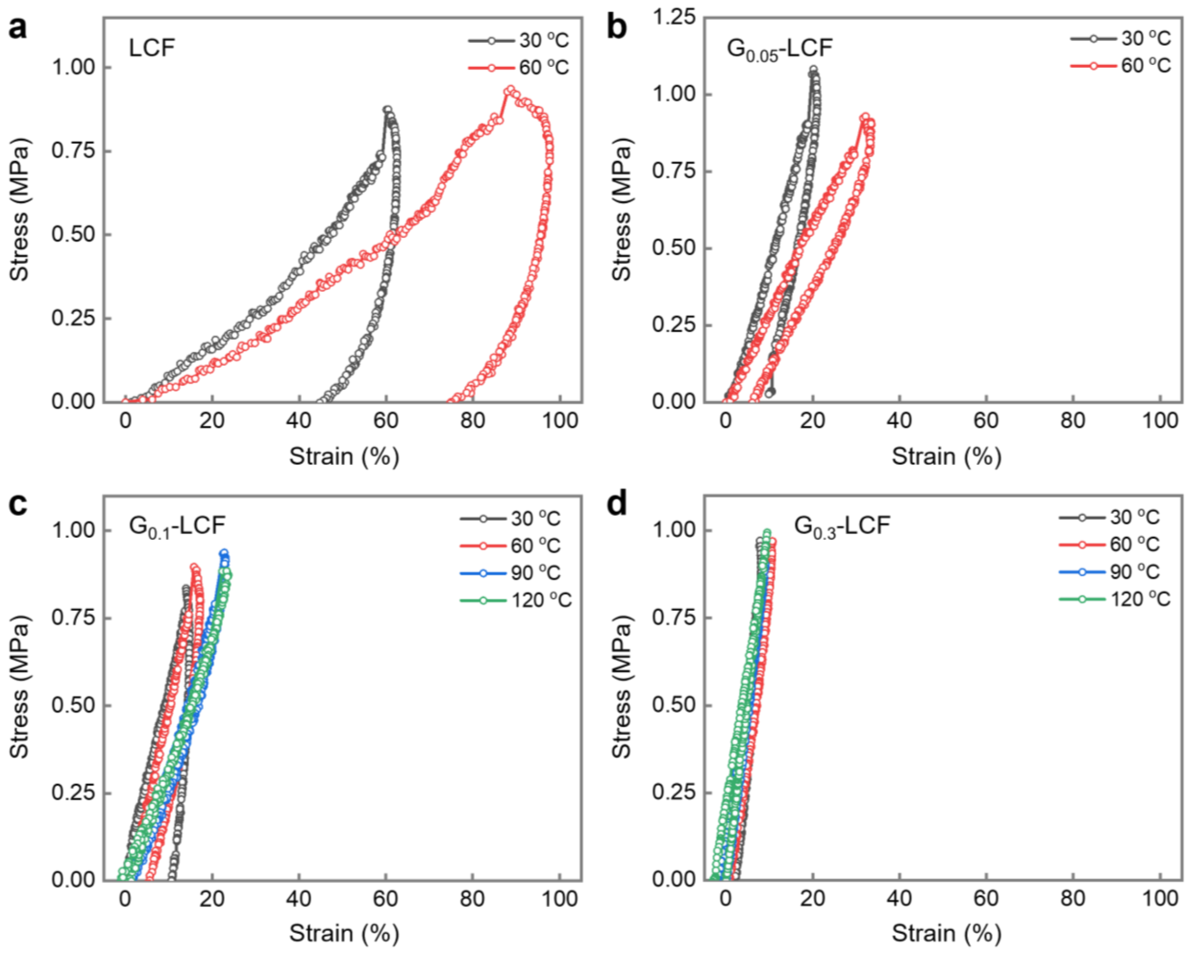

Supplementary Fig. 12 | Cyclic stress-strain curves of LCF and G-LCFs at different temperatures.

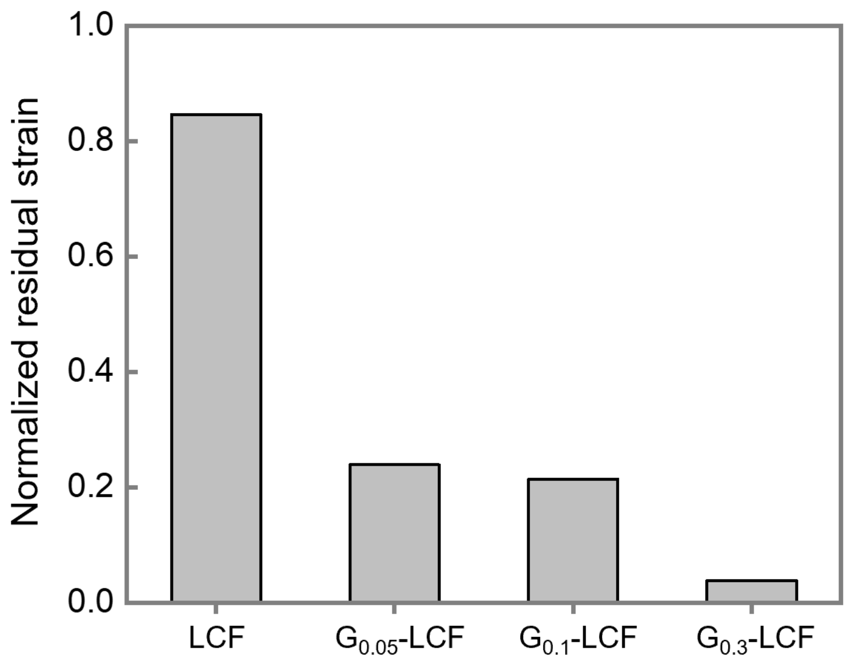

Supplementary Fig. 13 | Normalized residual strains of LCF and G-LCFs measured from uniaxial cyclic loading tests at 60 ºC.

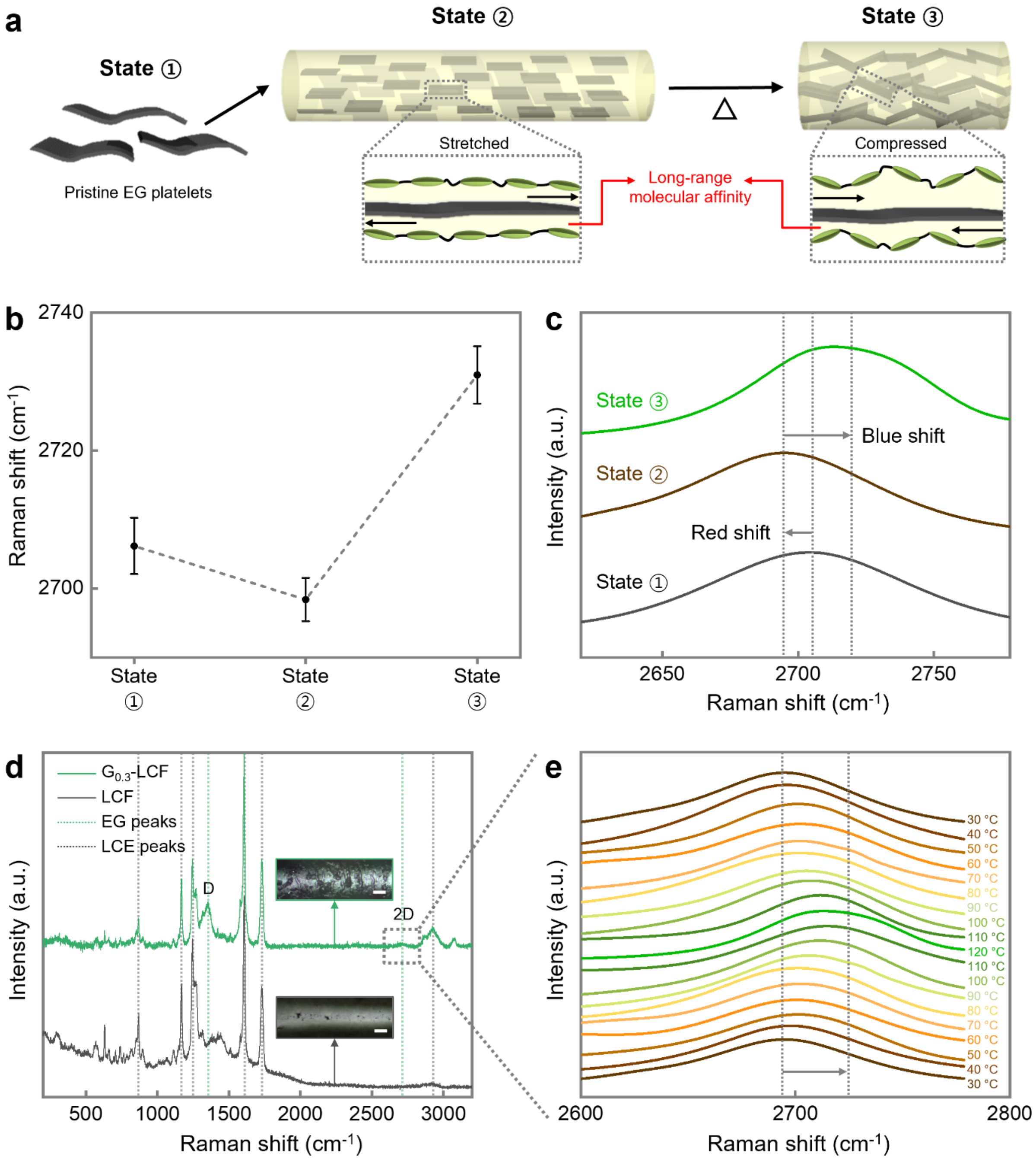

Supplementary Fig. 14 | a, Schematic illustration for the graphene 2D peak position; state 1: original pure graphene powder state, state 2: after composite actuator fiber spinning, and state 3: upon subsequent contractive actuation. **b, c,** Raman shift of graphene 2D peak upon fiber spinning and contractive actuation. **d,** Raman spectra of pure LCF and $G_{0.3}$-LCF. Scale bar, 50 μm. **e,** 2D band peaks of graphene fillers within $G_{0.3}$-LCF under temperature variation for contraction and recovery.

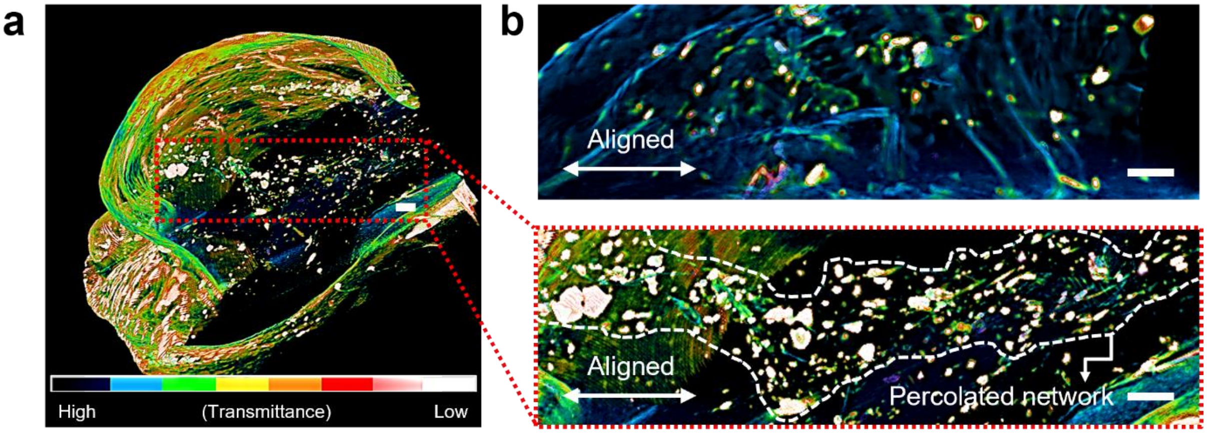

Supplementary Fig. 15 | a, XRM 3D mapping image of contracted $G_{0.3}$-LCF in terms of transmittance. **b,** Images for relaxed (up) and contracted (bottom) states of $G_{0.3}$- LCF. Scale bar, 20 μm.

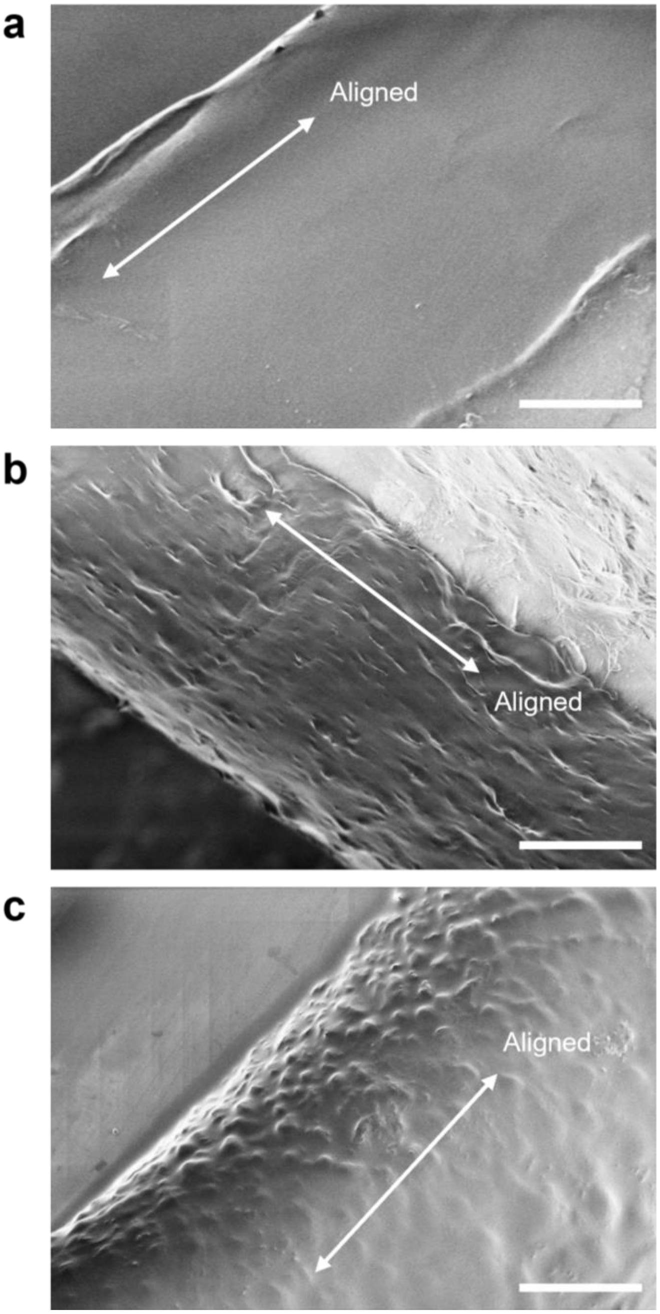

Supplementary Fig. 16 | SEM surface images of pure LCF (**a**), relaxed $G_{0.3}$-LCF (**b**), and contracted $G_{0.3}$-LCF (**c**). Scale bars, 10 μm (**a, b**), 50 μm (**c**).

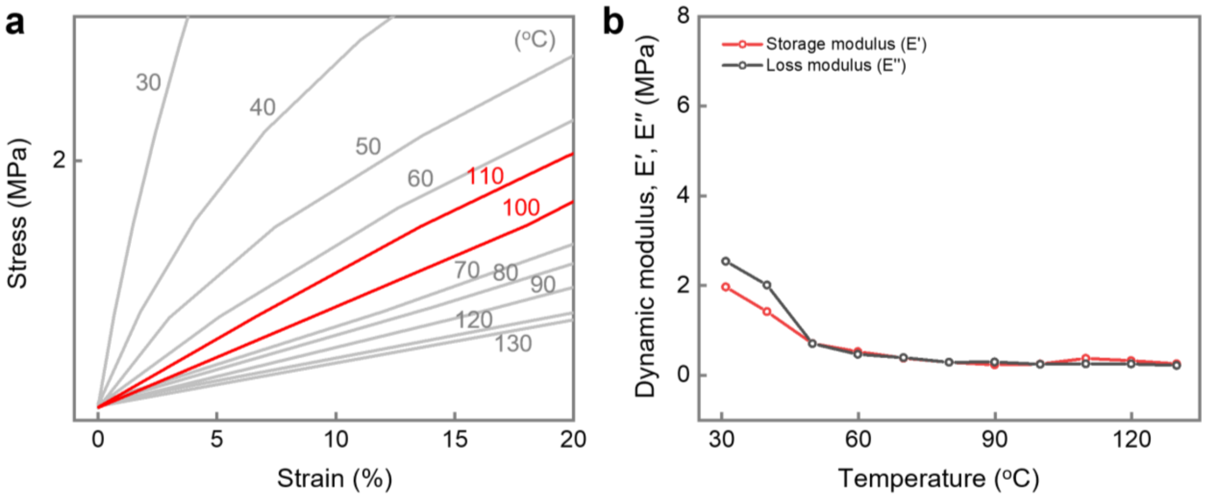

Supplementary Fig. 17 | a, Magnified stress-strain curves of $G_{0.3}$-LCF at low stress and strain levels at different temperatures. **b,** Variation of storage (E′) and loss (E″) moduli for pure LCF as a function of temperature.

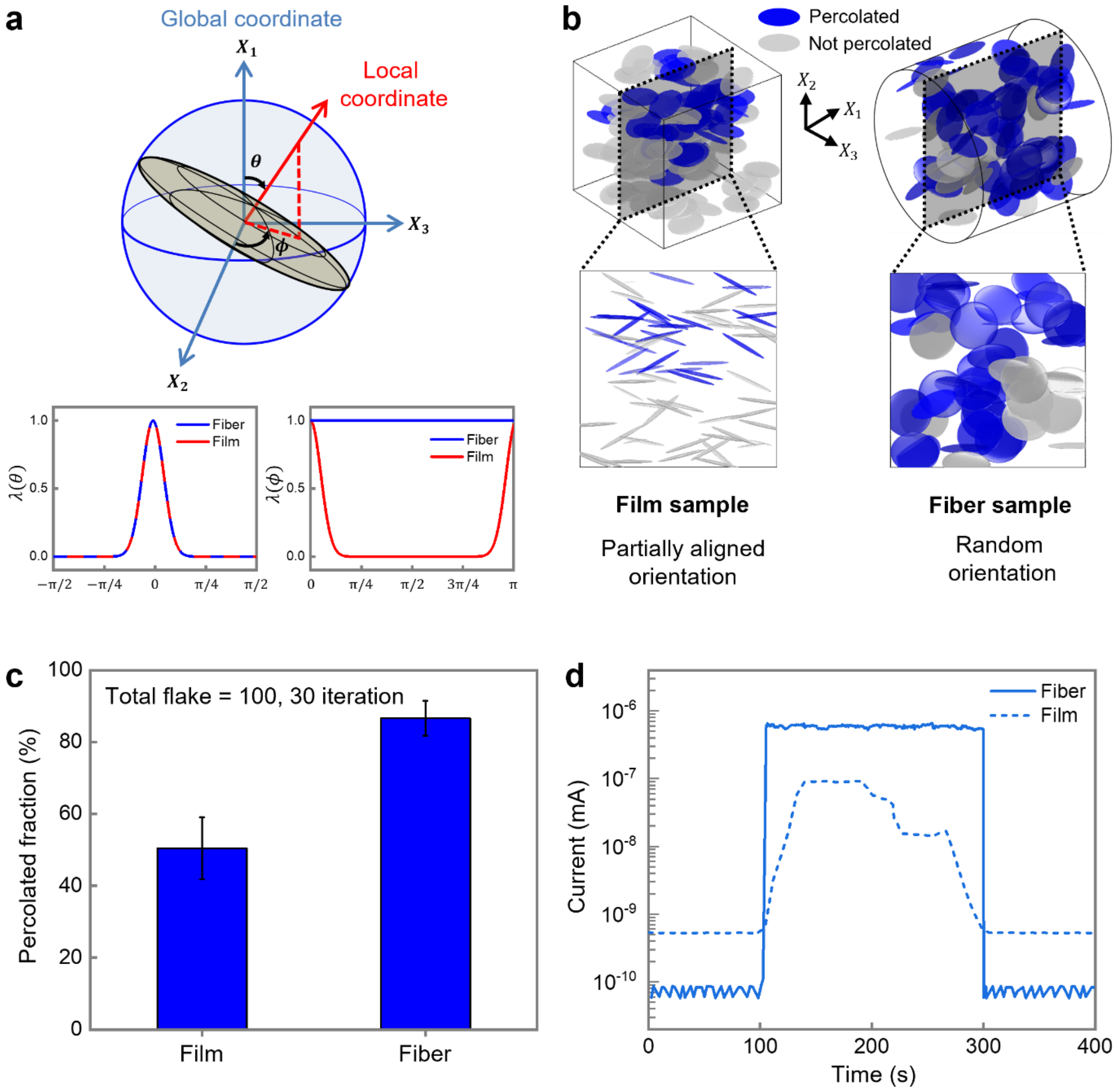

Supplementary Fig. 18 | a, Initial polar and azimuthal angle of platelets within film and fiber geometry composite. **b, c** Simulations for reversible percolation of EG flakes under contractive actuation (total flake of 100 and 30 iteration). **d,** Electrical current evolution of film and fiber type EG-LCE hybrid structure with 0.3 wt% EG during contractive actuation.

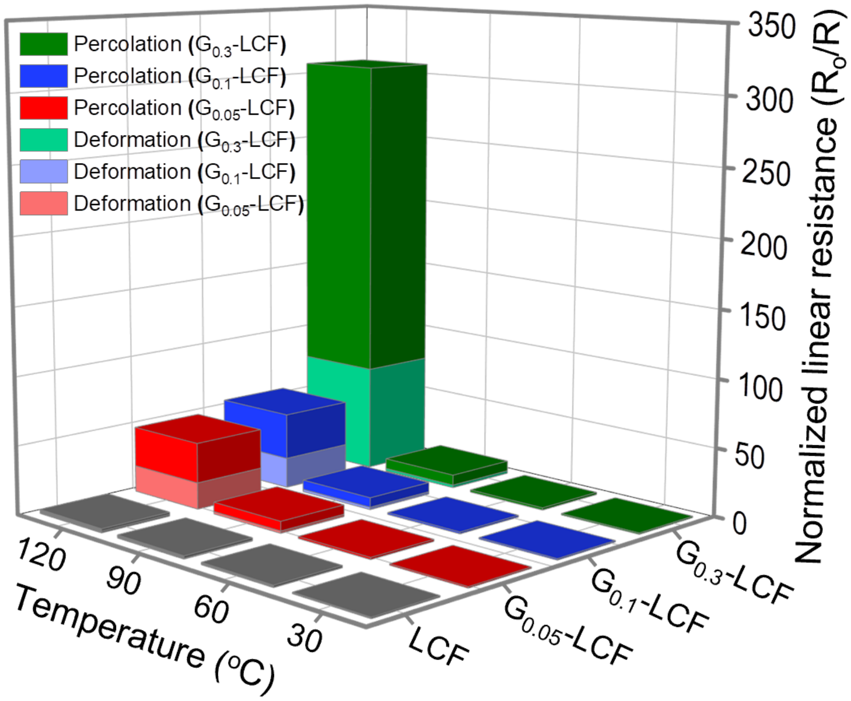

**Supplementary Fig. 19 | ** Normalized linear electrical resistance of LCF and G-LCFs at various temperatures. Distinguished resistance variations induced by reversible percolation and dimensional change.

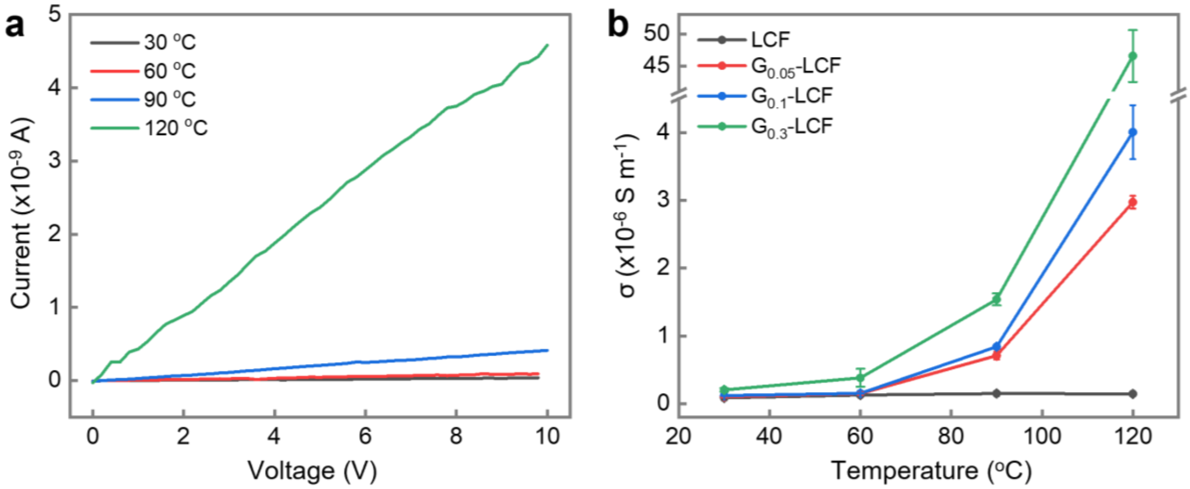

Supplementary Fig. 20 | a, I-V curves of $G_{0.3}$-LCF at different heating temperatures. **b,** Electrical conductivity of LCF and G-LCFs at various temperatures.

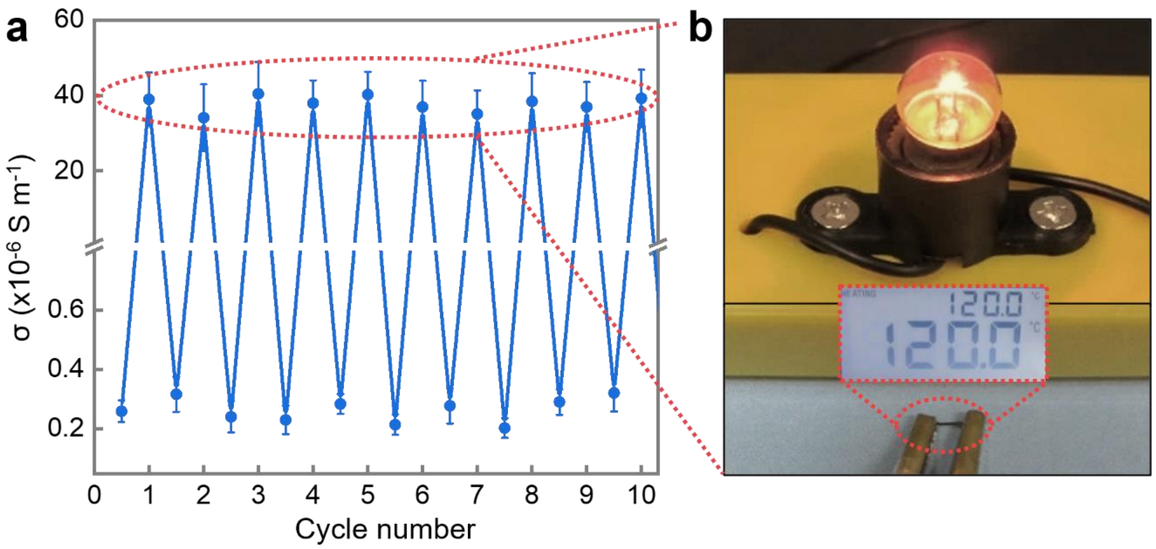

Supplementary Fig. 21 | a, Reversible change of electrical conductivity of $G_{0.3}$-LCF during reversible actuation over 10 cycles. **b,** Lightening candescent bulb at a voltage of 9 V while actuator fiber is under contraction at 120 ºC.

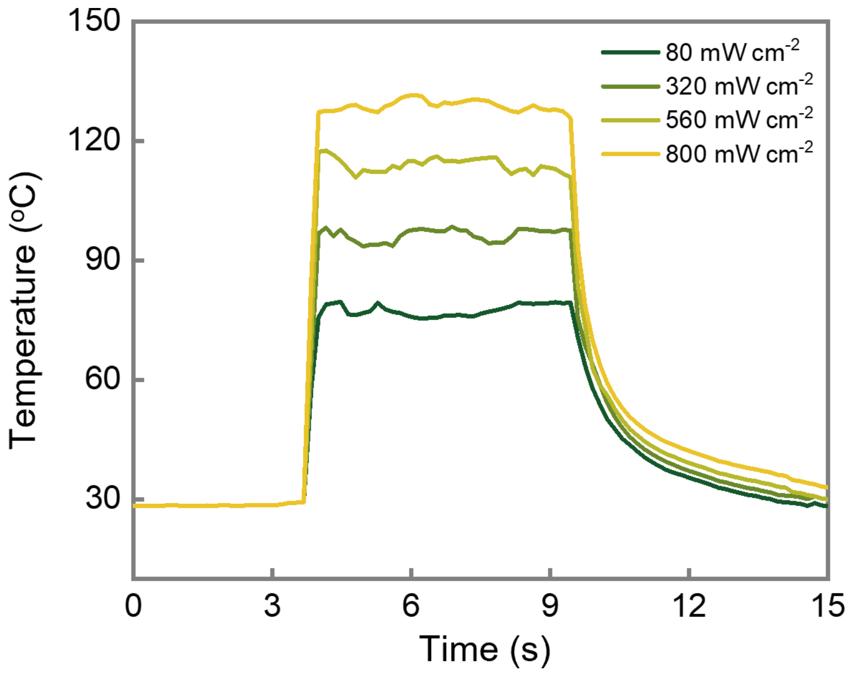

Supplementary Fig. 22 | Surface temperature variations of $G_{0.3}$-LCF (100-strands) under different NIR intensities.

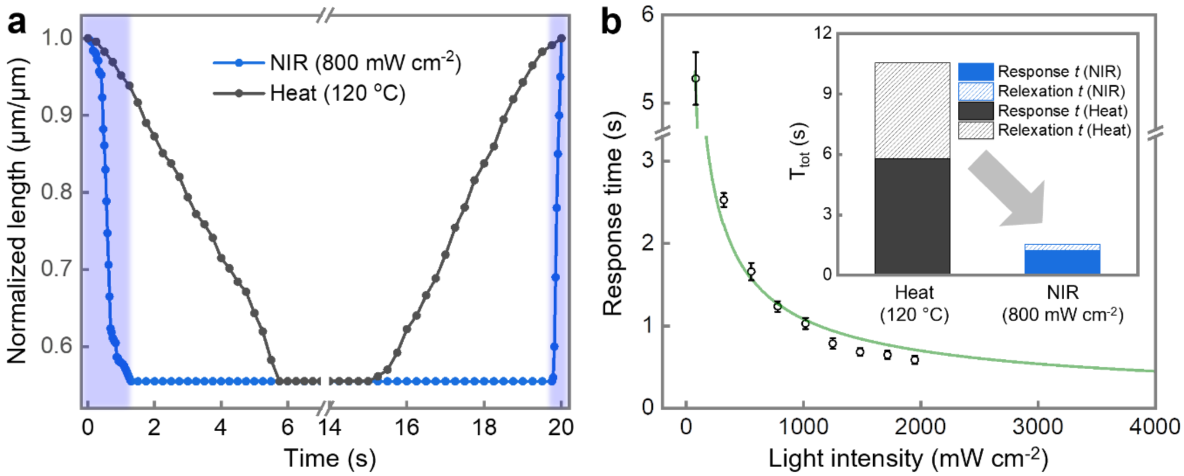

Supplementary Fig. 23 | a, Thermal and photothermal actuation of $G_{0.3}$-LCF in terms of time evolution of actuation behaviors. **b,** Response time of $G_{0.3}$-LCF as a function of NIR intensity, and comparison of response and relaxation time between thermally driven and light driven actuation (inset).

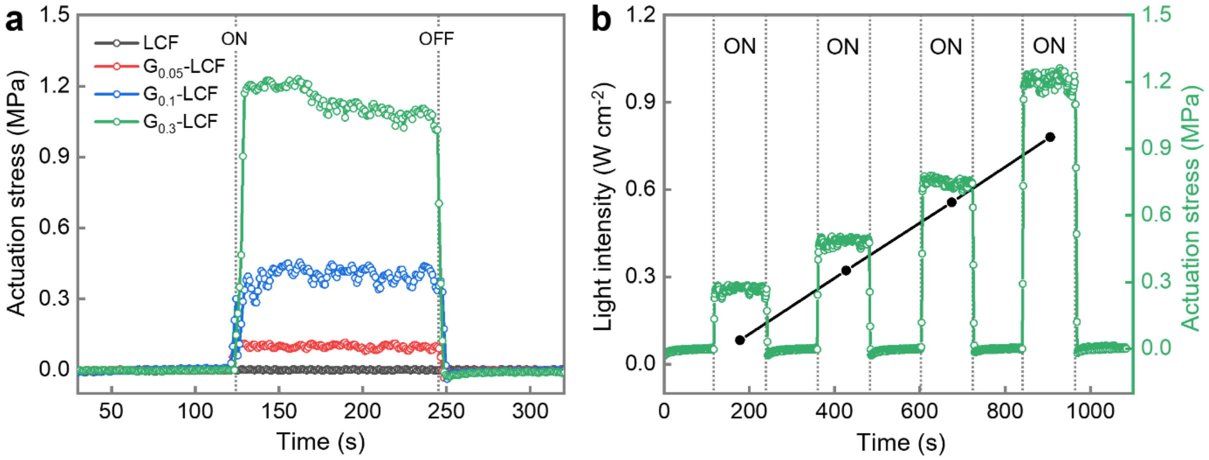

Supplementary Fig. 24 | a, Actuation stress generated from a single fiber level LCF and G-LCFs upon NIR irradiation (800 mW cm^{-2}). **b,** A single fiber level $G_{0.3}$-LCF at various NIR intensities under a constant strain (3%) at room temperature.

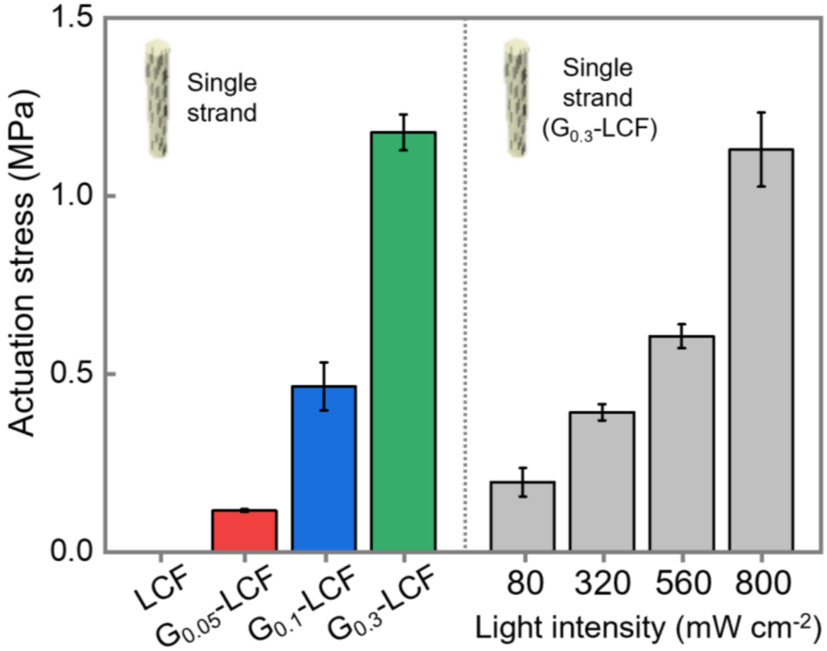

Supplementary Fig. 25 | Maximum actuation stress of single strand LCF and G-LCFs generated by NIR irradiation (800 mW cm^{-2}) (left), and photoactuation stress of a single strand $G_{0.3}$-LCF under various NIR intensities (right).

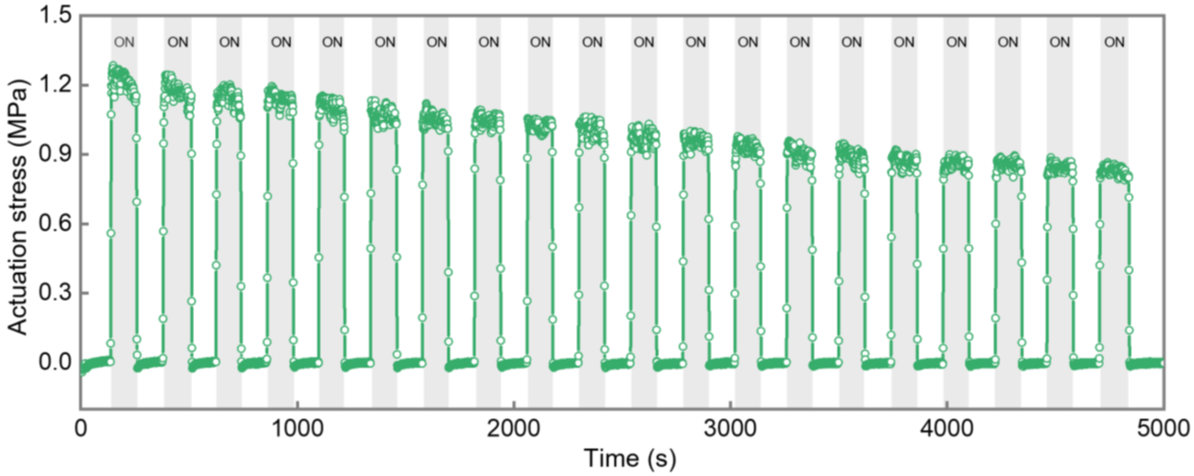

**Supplementary Fig. 26 | ** Long-term actuation stress generation test of $G_{0.3}$-LCF in single fiber level upon NIR control.

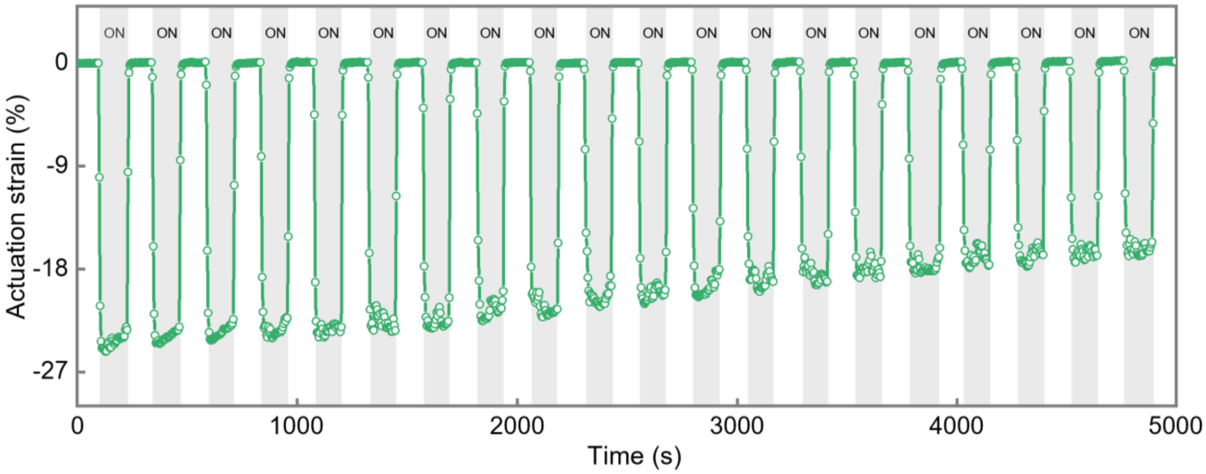

Supplementary Fig. 27 | Long-term actuation strain contraction test of $G_{0.3}$-LCF in single fiber level upon NIR control.

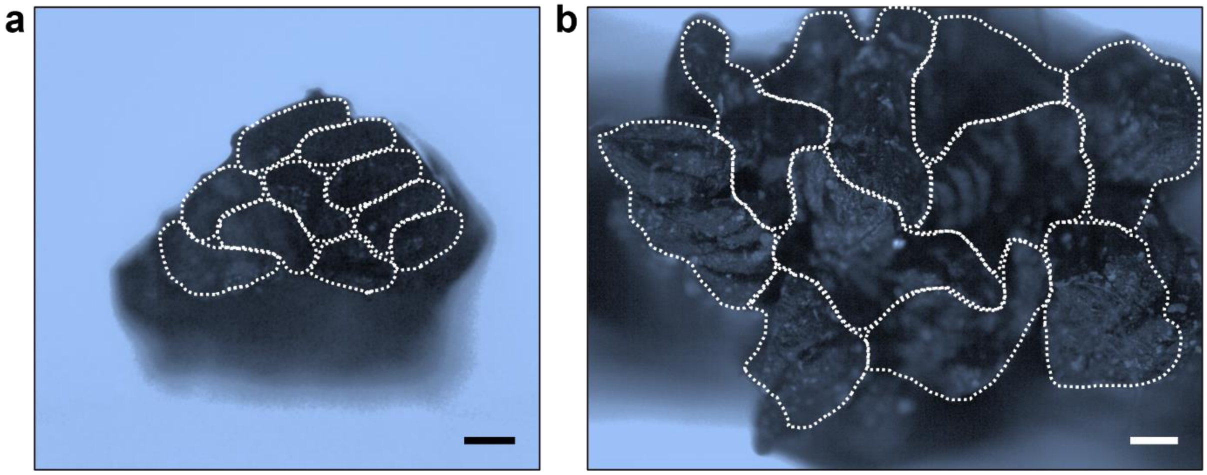

Supplementary Fig. 28 | Cross-sectional SEM images of $G_{0.3}$-LCF bundle consisting of 10-strands (**a**) and 100-strands (**b**). Scale bars, 100 μm (Individual circle indicates a single strand fiber) (**a**), 200 μm (Individual circle indicates 10-strands fiber) (**b**).

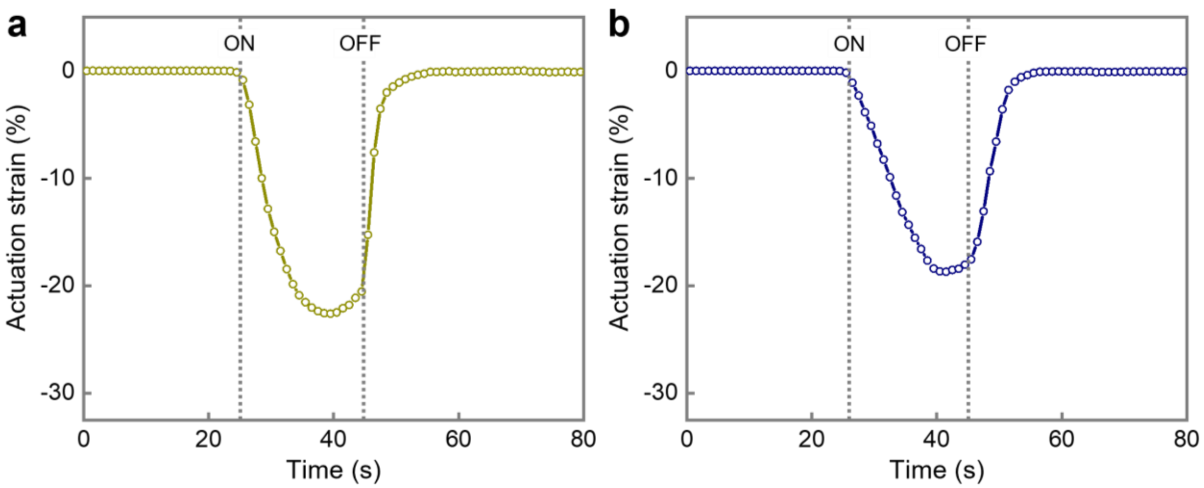

Supplementary Fig. 29 | ** Contraction strain in response to NIR irradiation (800 mW cm^{-2}) of 10-strand bundle (a**) and 100-strand bundle (**b**) $G_{0.3}$-LCF under a constant stress (3 MPa).

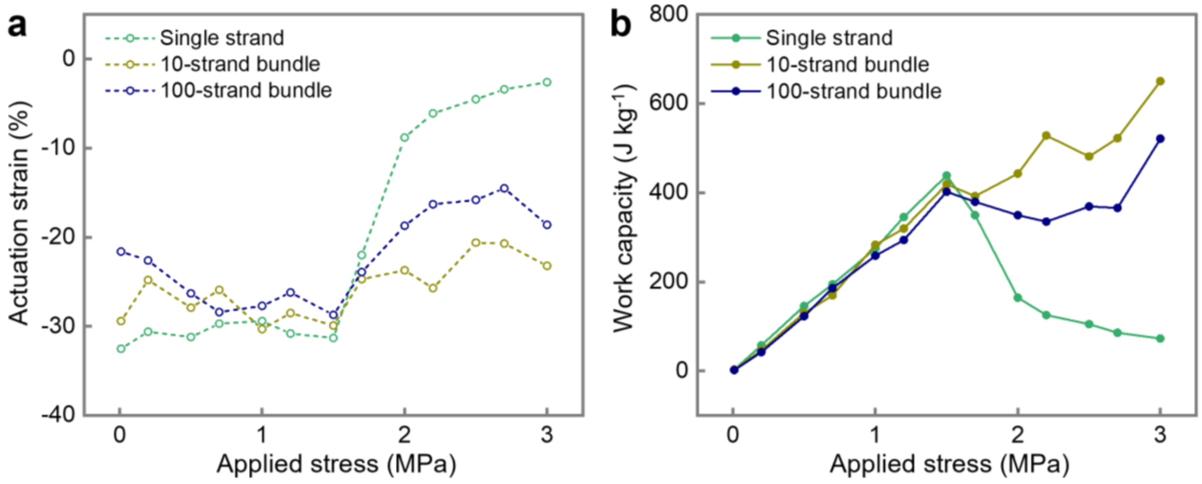

Supplementary Fig. 30 | Evolution of maximum contraction strain (**a**) and work capacity (**b**) for different strands of $G_{0.3}$-LCF against various applied stress upon NIR irradiation (800 mW cm^{-2}).

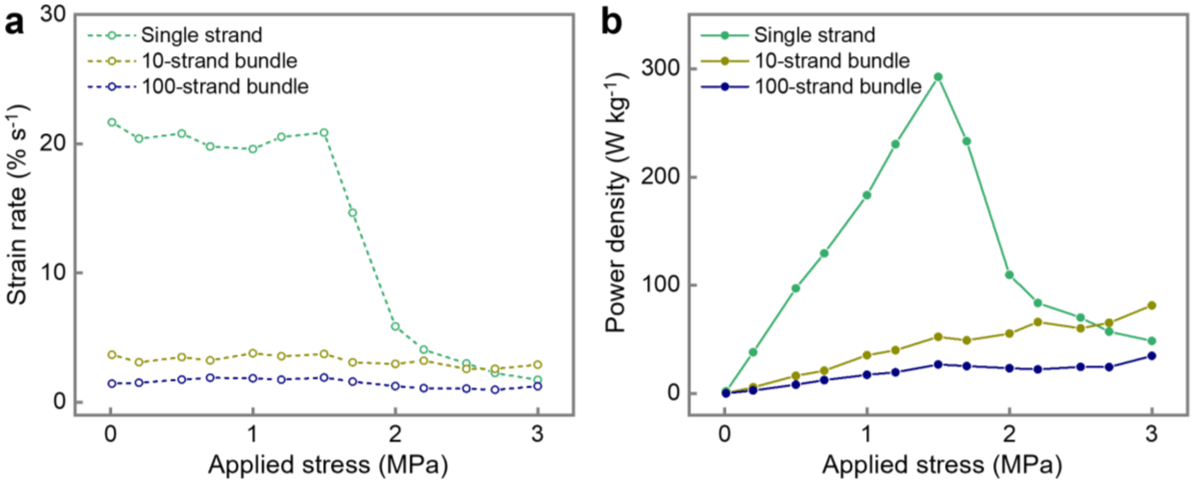

Supplementary Fig. 31 | Strain rate (**a**) and power density (**b**) of single strand, 10-strand bundle, and 100-strand bundle of $G_{0.3}$-LCF upon NIR irradiation (800 mW cm^{-2}) as a function of applied stress.

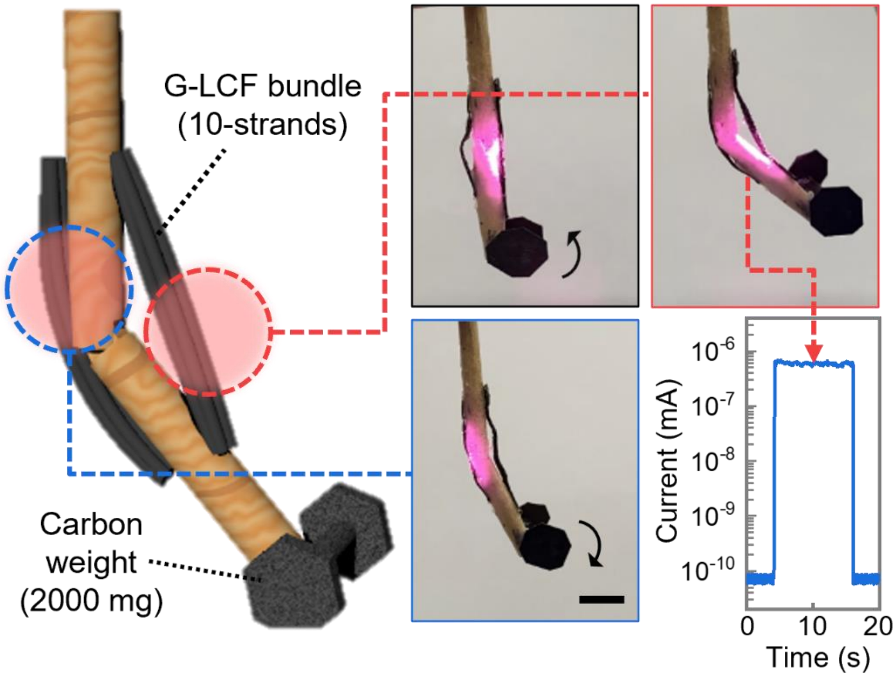

Supplementary Fig. 32 | Artificial arm under NIR remote photothermal manipulation along with electrical current variation. Scale bar, 1cm.

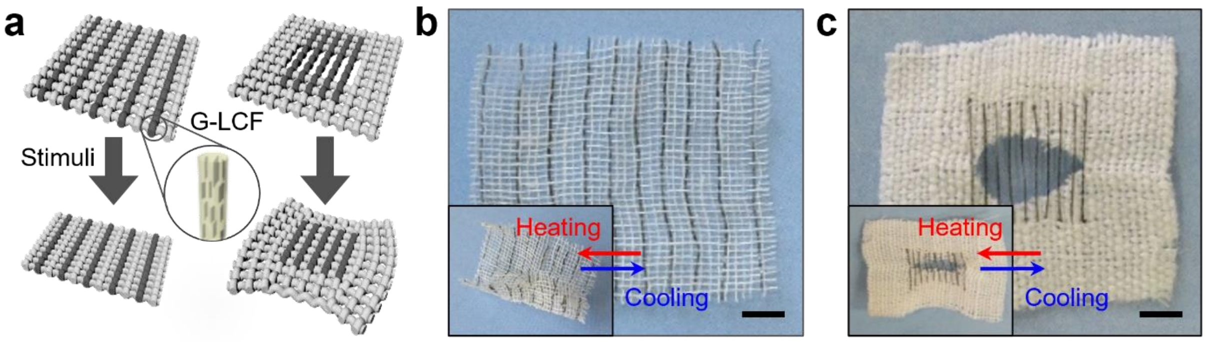

Supplementary Fig. 33 | a, Schematic of smart textile woven with G-LCFs for programmable shape deformation. **b, c** Images of reversible thermal actuation of smart textiles. Scale bar: 1 cm.

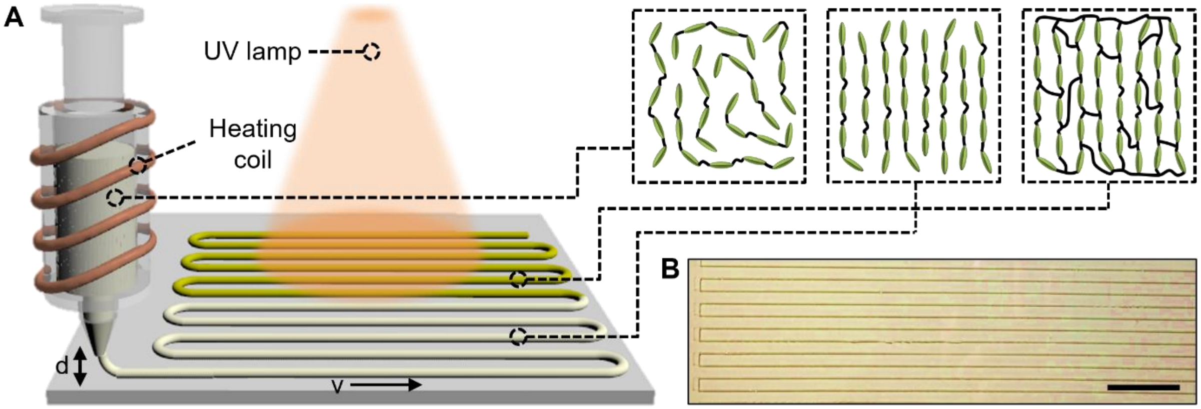

Supplementary Fig. 34 | a, Schematic illustration of direct melt-spinning of LCE-based fibers and the configuration of LC mesogenic units at each processing step. **b,** Resultant continuous fibers on collector. Scale bar, 5 cm.

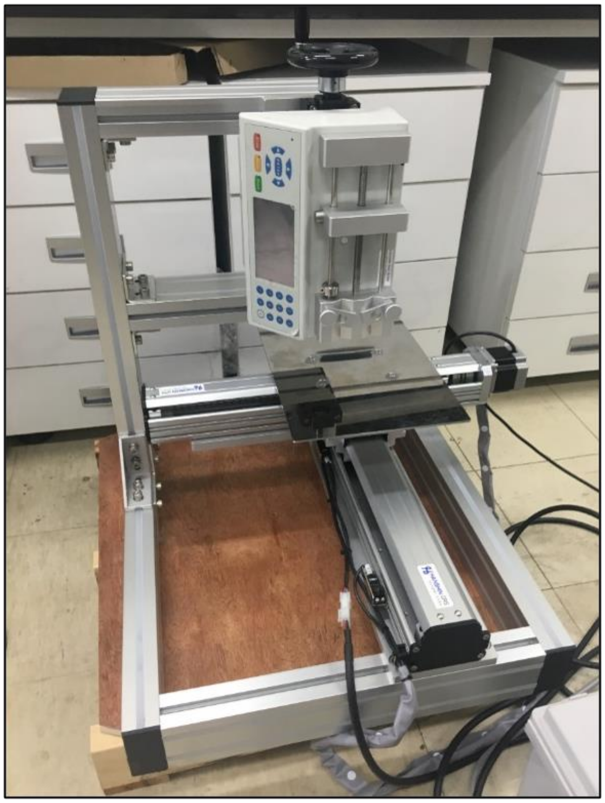

Supplementary Fig. 35 | Photograph of homemade customized equipment for direct melt-spinning of LCE–based fibers.

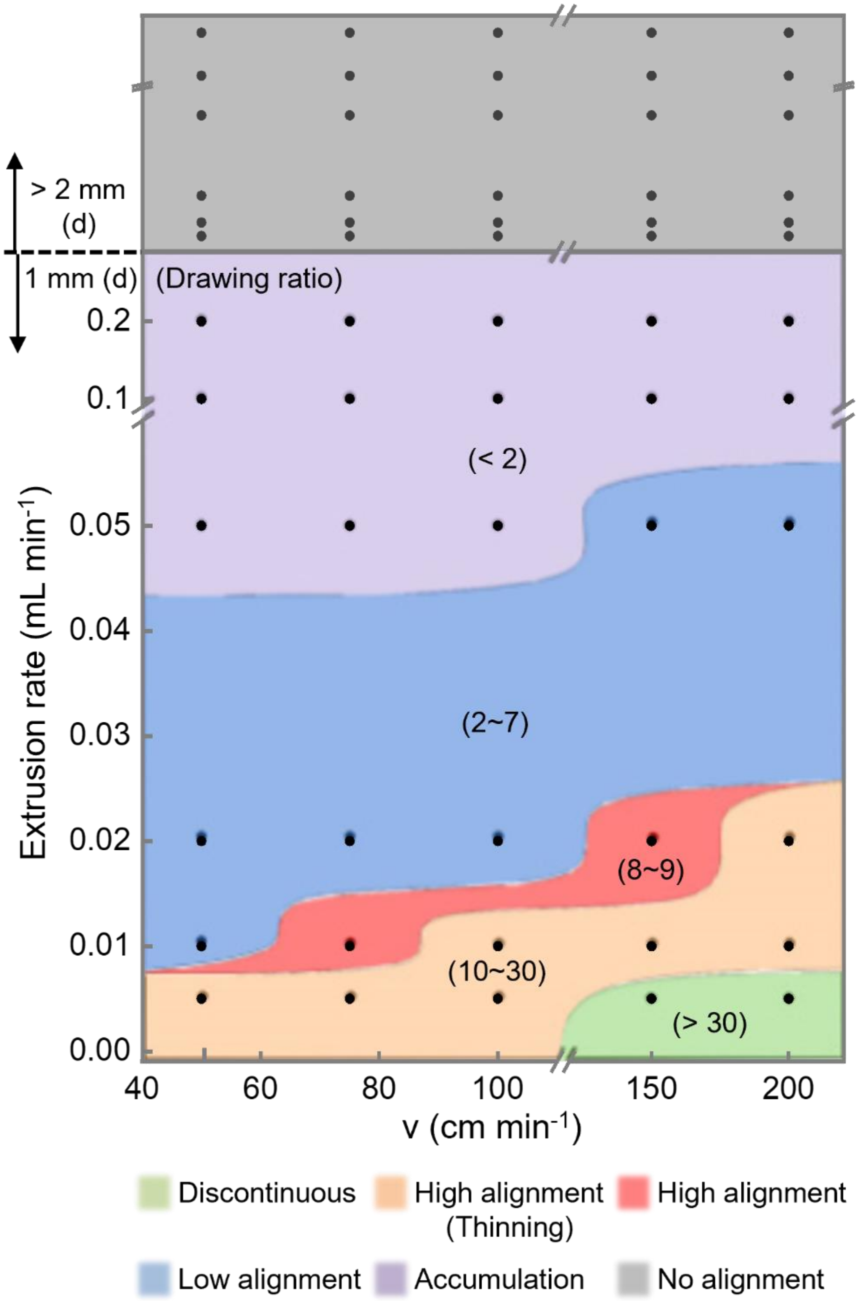

Supplementary Fig. 36 | Fiber spinnability and alignment diagram based on the drawing ratios of spun fibers.

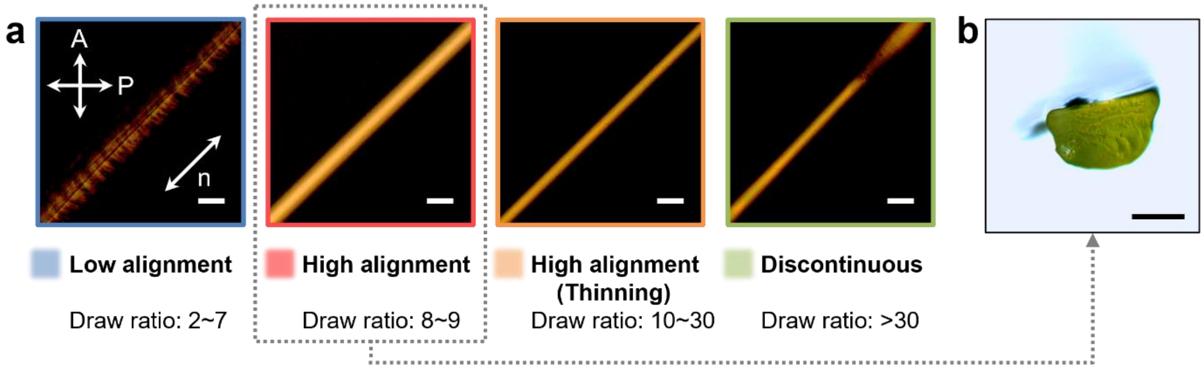

Supplementary Fig. 37 | a, POM images of LCFs observed at 45° with respect to cross polarizers at different drawing ratios. **b,** Optical microscopy observation of cross-section of resultant highly aligned LCF. Scale bars, 200 μm (**a**), 100 μm (**b**).

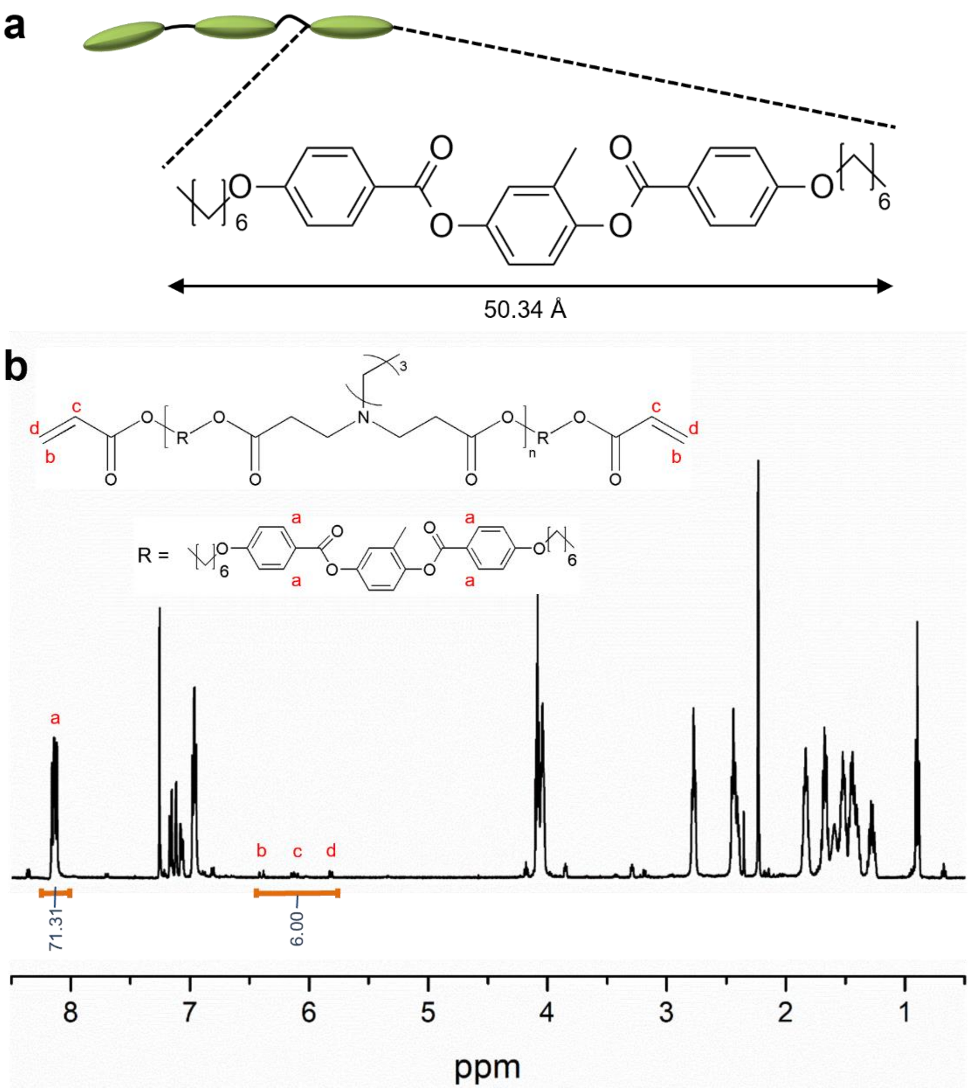

Supplementary Fig. 38 | a, Chemical structure of individual nematic monomer. **b,** ^{1}H NMR spectrum of LC oligomer in $CDCl_3$.

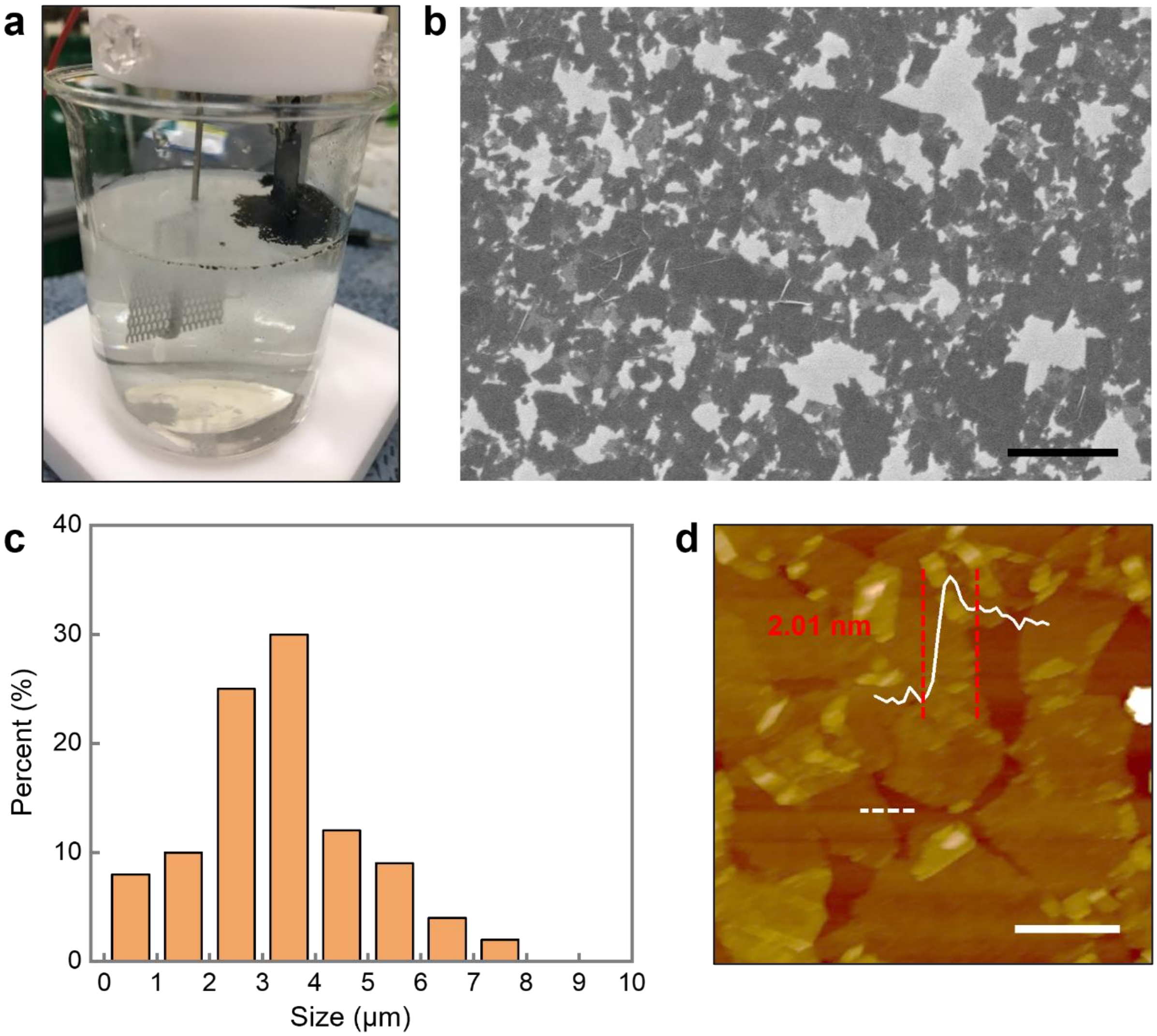

Supplementary Fig. 39 | a, Photograph of electrochemically exfoliation of graphite foil. **b,** SEM image of EG flakes. **c,** Lateral size distribution of EG flakes. **d,** AFM image with height profile of EG flakes. Scale bars, 5 µm (**b**), 1 µm (**d**).

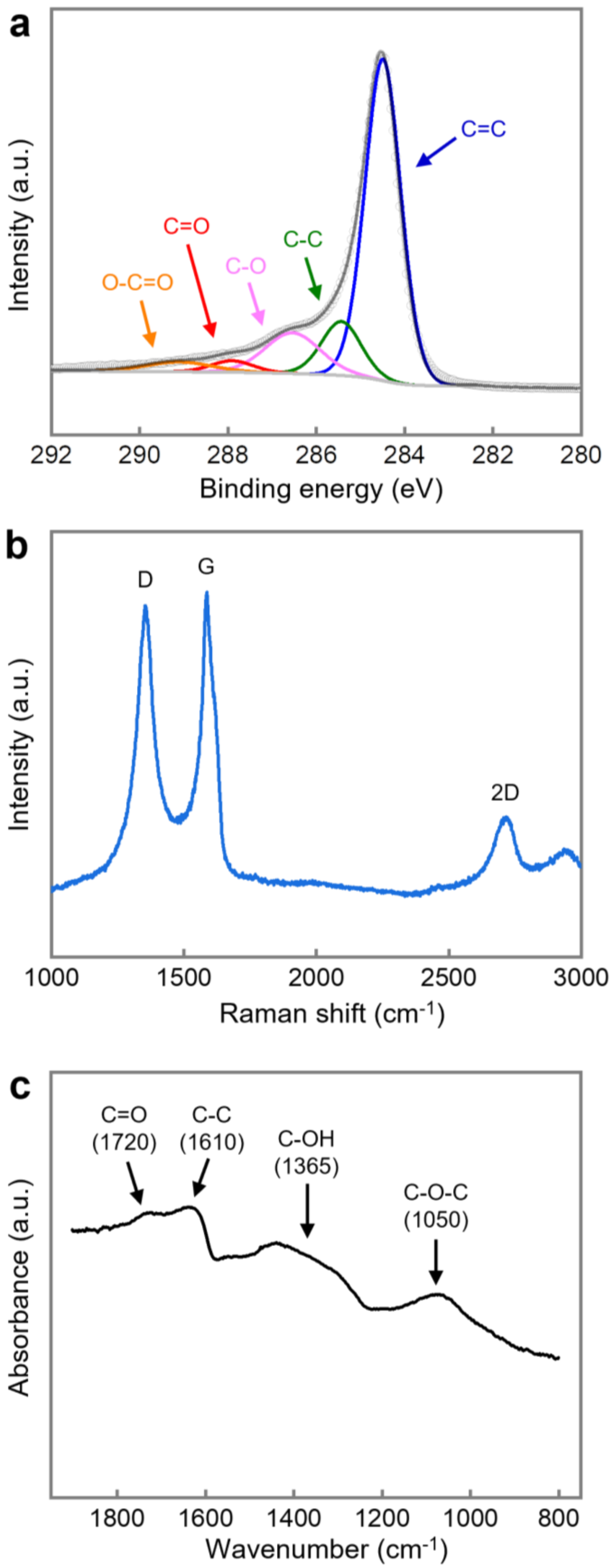
a
Intensity (a.u.)
C=C
C=O
C-C
C-O
O-C=O
292
290
288
286
284
282
280
Binding energy (eV)
b
Intensity (a.u.)
D
G
2D
1000
1500
2000
2500
3000
Raman shift (cm-1)
c
Absorbance (a.u.)
C=O
(1720)
C-C
(1610)
C-OH
(1365)
C-O-C
(1050)
1800
1600
1400
1200
1000
800
Wavenumber (cm-1)

Supplementary Fig. 40 | a, XPS spectra at C1s region. **b,** Raman spectrum with D, G, 2D peak. **c,** FT-IR spectra in the wavenumber from 800 to 1800 cm^{-1} of EG.

	LCF	$G_{0.05}$-LCF	$G_{0.1}$-LCF	$G_{0.3}$-LCF (Single-strand)	$G_{0.3}$-LCF (10-strands)	$G_{0.3}$-LCF (100-strands)
Diameter (μm)	203	203	203	203	592	2714
Young's modulus (MPa)	12.8	33.4	38.8	86.1	-	-
Actuation stress (MPa)	-	0.12	0.55	1.23	-	-
Minimum response time (s)	5.7	0.9	0.8	0.5	2.3	5
Aactuation strain (%)	47	45	45	45	45	45
Specific actuation stress (Nm/kg)	-	113	517	1153	-	-
Actuation strain rate (%/s)	8.2	51.1	57.7	90	19.5	9
Work capacity (J/kg)	-	-	-	439	650	522
Power density (W/kg)	-	-	-	293	81	35

Supplementary Table 1 | Specifications and performances (in maximum values) of our artificial muscle fiber with different EG loading amount in elastomeric matrix and recombinant structures.

Actuation stress (MPa)	Work capacity (J/kg or kJ/m^3)	Reference
–	9.2 (J/kg)	[77]
–	38 (J/kg)	[78]
0.23	0.78 (kJ/m^3)	[79]
0.08	2.7 (kJ/m^3)	[80]
0.11	10 (kJ/m^3)	[81]
–	20 (kJ/m^3)	[82]
–	40 (kJ/m^3)	[68]
0.4	86 (kJ/m3)	[4]
0.35	150 (kJ/m^3)	[83]
0.28	180 (kJ/m^3)	[84]
–	262 (kJ/m^3)	[85]
1.3	730 (kJ/m3)	[86]
2.53	1267 (kJ/m^3)	[76]
0.35	40 (J/kg)	Human muscle [58]
**1.23**	**650 (J/kg), 695.5 (kJ/m^3)**	**This work**

Supplementary Table 2 | Comparison of actuation stress and work capacity among previous LCE actuators, human muscle, and this work.

Actuation Strategy	Speed (mm/s)	Reference
Pneumatic/hydraulic	8.75	[87]
	5.14	[88]
Dielectric elastomer	1.95	[89]
	3.13	[90]
Liquid cryatal elastomer	0.03	[91]
	0.24	[92]
Twisted and coiled polymer	1.2	[93]
Magnetoactive material	0.5	[94]
Natural inchworm	2.5	-
**LCE-graphene composite**	**7.2**	**This work**

Supplementary Table 3 | Comparison of crawling speed of this work with those of previously reported soft crawlers based on different actuation mechanisms.
